# Synthesis, crystal structure and properties of μ-tetra­thio­anti­monato-bis­[(cyclam)zinc(II)] perchlorate 0.8-hydrate

**DOI:** 10.1107/S2056989024009356

**Published:** 2024-10-11

**Authors:** Christian Näther, Henning Lühmann, Wolfgang Bensch

**Affiliations:** a Institut für Anorganische Chemie, Universität Kiel, Max-Eyth.-Str. 2, D-24118 Kiel, Germany; University of Aberdeen, United Kingdom

**Keywords:** crystal structure, zinc thio­anti­monate, layered structure, hydrogen bonding

## Abstract

The reaction of Zn(ClO_4_)_2_·6H_2_O with Na_3_SbS_4_·9H_2_O in a water/aceto­nitrile mixture leads to the formation of the title compound, (μ-tetra­thio­anti­monato-κ^2^*S*:*S*′)bis­[(1,4,8,11-tetra­aza­cyclo­tetra­decane-κ^4^*N*)zinc(II)] perchlorate 0.8-hydrate, [Zn_2_(SbS_4_)(C_10_H_24_N_4_)_2_]ClO_4_·0.8H_2_O or [(Zn-cyclam)_2_(SbS_4_)]^+^[ClO_4_]^−^·0.8H_2_O. The asymmetric unit consists of two crystallographically independent [SbS_4_]^3–^ anions, two independent perchlorate anions and two independent water mol­ecules as well as four crystallographically independent Zn(cyclam)^2+^ cations that are located in general positions. Both perchlorate anions and one cyclam ligand are disordered and were refined with a split mode using restraints. The water mol­ecules are partially occupied. Two Zn(cyclam)^2+^ cations are linked *via* the [SbS_4_]^3–^ anions into [Zn_2_(cyclam)_2_SbS_4_]^+^ cations that are charged-balanced by the [ClO_4_]^−^ anions. The water mol­ecules of crystallization are hydrogen bonded to the [SbS_4_]^3–^ anions. The cations, anions and water mol­ecules are linked by N—H⋯O, N—H⋯S and O—H⋯S hydrogen bonds into a three-dimensional network. Powder X-ray diffraction proves that a pure sample had been obtained that was additionally investigated for its spectroscopic properties.

## Abstract 2

In the title compound, the [SbS_4_]^3–^ anions bridge two Zn(cyclam)^2+^ cations into [Zn_2_(cyclam)_2_SbS_4_]^+^ cations, which are charged-balanced by perchlorate anions. The components are linked by N—H⋯O, N—H⋯S and O—H⋯S hydrogen bonds into a three-dimensional network.

## 1. Chemical context

Thio­anti­monate(III) compounds show a large structural variability, which in part can be traced back to the free lone-electron pair of Sb^3+^. Their structural chemistry is characterized by SbS_*x*_ anions that can condense into larger anions such as rings, chains or layers (Sheldrick & Wachhold, 1998 ▸; Zhou, 2016 ▸; Zhu & Dai, 2017 ▸; Bensch *et al.*, 1997 ▸; Spetzler *et al.*, 2004 ▸; Puls *et al.*, 2006 ▸; Lichte *et al.*, 2009 ▸). Charge balance is frequently achieved by inorganic or organic cations. Some of such compounds also have potential for applications as, for example, photoconductive materials (Pienack *et al.*, 2008*a* ▸) or in the field of superionic conductors (Zhou *et al.*, 2019 ▸) and this is one reason why we have been inter­ested in such compounds for many years (Schaefer *et al.*, 2003 ▸; Schur *et al.*, 1998 ▸; 2001 ▸; Stähler *et al.*, 2001 ▸; Kiebach *et al.*, 2004 ▸; Lühmann *et al.*, 2008 ▸; Pienack *et al.*, 2008*b* ▸; Engelke *et al.*, 2004 ▸, 2008 ▸). In contrast to these compounds, the chemistry of thio­anti­monates(V) with Sb^5+^ is less developed, and in most cases the cations and [SbS_4_]^3–^ anions are separated (Schur *et al.*, 1998 ▸; Jia *et al.*, 2004 ▸; Kiebach *et al.*, 2004 ▸; Wang *et al.*, 2013 ▸). In only a few structures are the [SbS_4_]^3–^ anions and cations found to be connected (Jia *et al.*, 2005 ▸; Danker *et al.*, 2021 ▸; Näther *et al.*, 2022 ▸).

Concerning the synthesis of thio­anti­monate compounds, in the majority of examples solvothermal synthesis starting from the elements was used. Later we found that elemental anti­mony and sulfur can be replaced by NaSbS_3_ or by Schlippe’s salt (Na_3_SbS_4_·9H_2_O; Anderer *et al.*, 2014 ▸, 2016*a* ▸). Schlippe’s salt is unstable and forms different reactive species and a variety of complex redox and condensation reactions can occur leading to the formation of thio­anti­moante(III) species (Rammelsberg, 1841 ▸; Long *et al.*, 1970 ▸; Mosselmanns *et al.*, 2000 ▸; Planer-Friedrich & Scheinost, 2011 ▸; Planer-Friedrich & Wilson, 2012 ▸). Later we found that for the directed synthesis of thio­anti­monates(III) the reaction temperature must be reduced, which is accompanied by a slower decomposition of Schlippe’s salt. This is an advantage, because such compounds can be prepared at room-temperature (Anderer *et al.*, 2016*b* ▸; Hilbert *et al.*, 2017 ▸).

However, as mentioned above, the synthesis of thio­anti­monate(V) compounds that are linked to transition metal cations is still not easy but can be achieved by using tetra­dentate ligands that, in an octa­hedral coordination of a transition-metal catio,n provide two coordination sites for bond formation to the thio­anti­monate(V) anions. In this context, cyclam (1,4,8,11-tetra­aza­cyclo­tetra­decane, C_10_H_24_N_4_) is a promising ligand. Following this consideration we reported on two new polymeric thio­anti­monates(V) with the compositions [(Cu-cyclam)_3_(SbS_4_)_2_]·20H_2_O and [(Zn-cyclam)_3_(SbS_4_)_2_]·8H_2_O (Danker *et al.*, 2021 ▸), in which the metal cations are linked to the [SbS_4_]^3–^ anions. Later we prepared a similar compound with cobalt that forms thio­anti­monate layers, as it was previously the case for the Cu compound (Näther *et al.*, 2022 ▸). In contrast to the Cu and Co cyclam compounds, no layers were formed in [(Zn-cyclam)_3_(SbS_4_)_2_]·8H_2_O because the Zn cations are disordered over both N_4_ planes of the cyclam ligands (Danker *et al.*, 2021 ▸). In the course of this project we obtained crystals of the new title compound with the composition [(Zn-cyclam)_2_(SbS_4_)]^+^[ClO_4_]^−^·0.8H_2_O, (I), which was characterized by single crystal X-ray diffraction and powder X-ray diffraction as well as by IR and Raman spectroscopy.
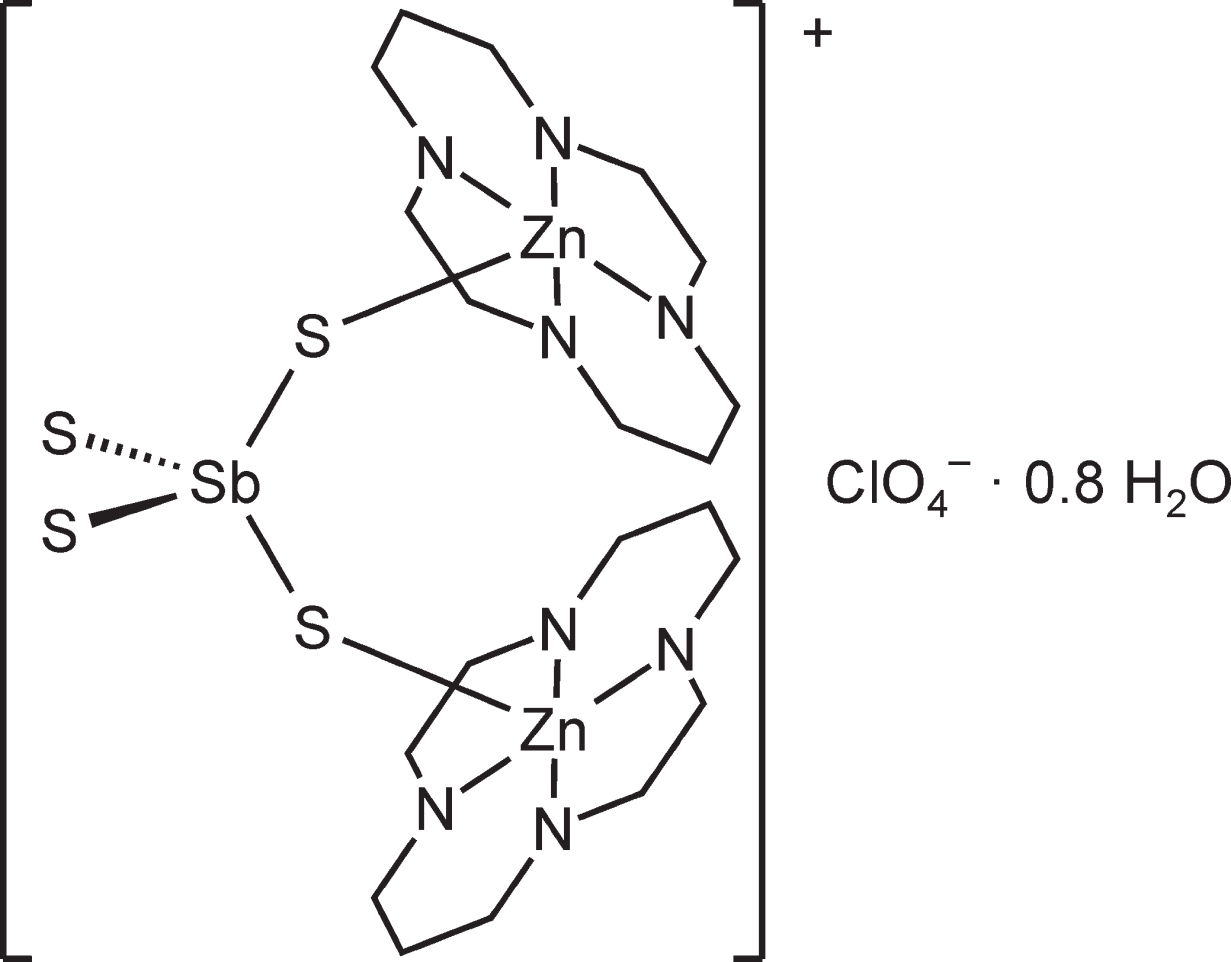


## 2. Structural commentary

**Figure 1:**
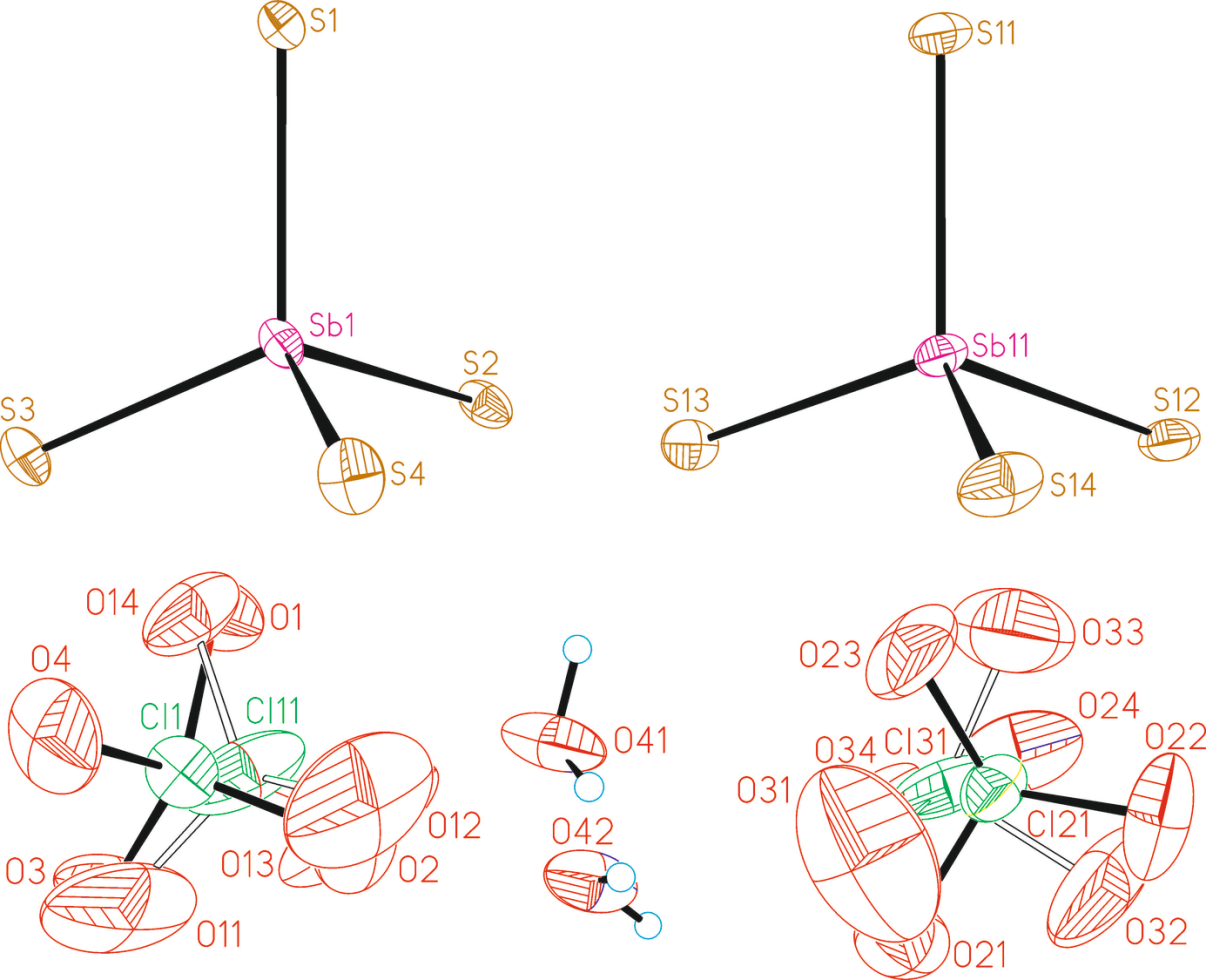
The tetra­thio­anti­monate and perchlorate anions and water mol­ecules in (I) with displacement ellipsoids drawn at the 50% probability level. The disorder of the perchlorate anions is shown with full and open bonds.

**Figure 3:**
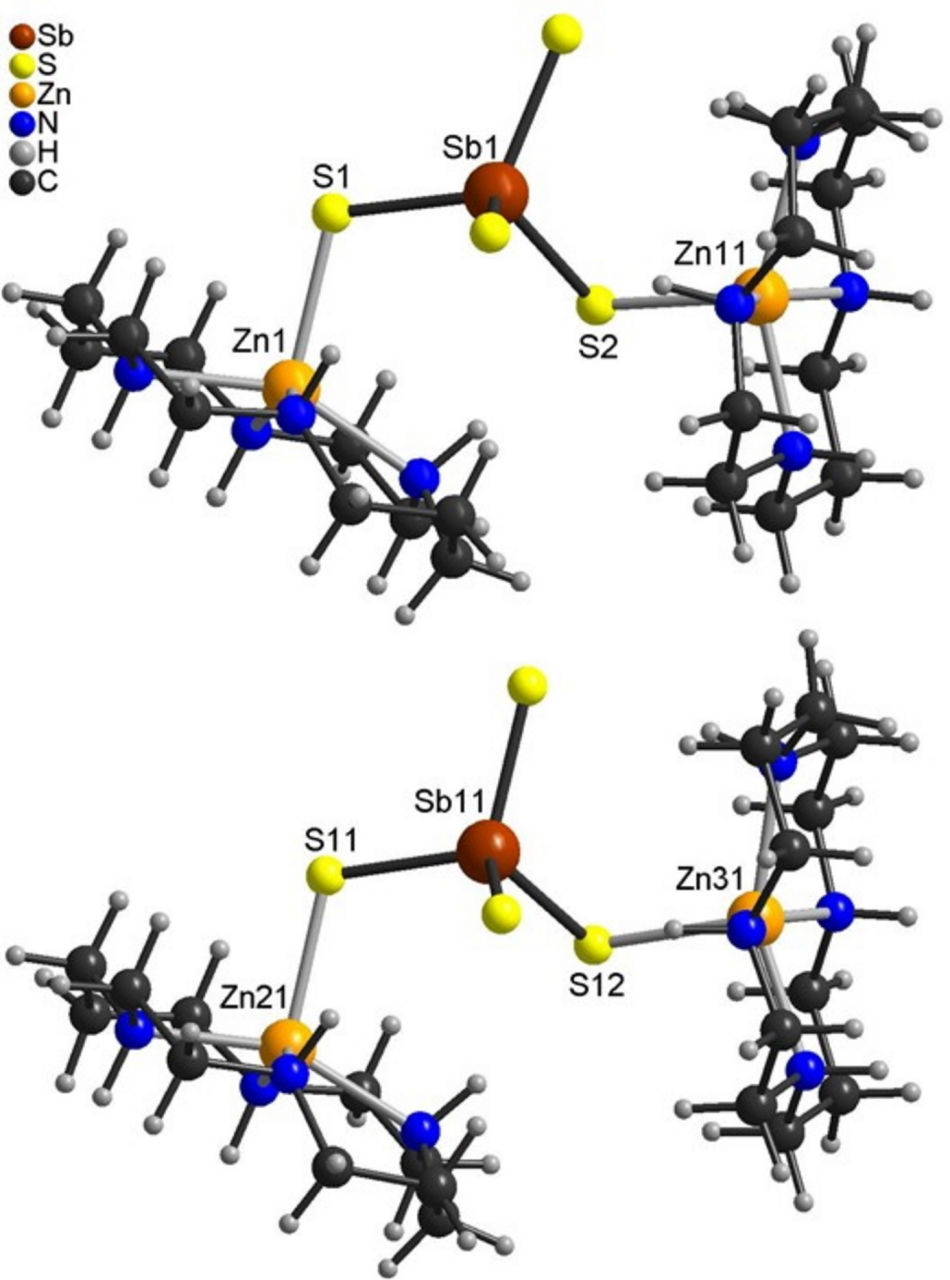
View of the Zn(cyclam)^2+^–[SbS_4_]^3–^–Zn(cyclam)^2+^ units in (I).

The asymmetric unit of (I) consists of four crystallographically independent Zn(cyclam)^2+^ cations as well as two crystallographically independent [SbS_4_]^3–^ anions, two perchlorate anions and two water mol­ecules that are located in general positions (Figs. 1 ▸ and 2 ▸). Each of the four crystallographically independent Zn^2+^ cations is coordinated by the four N atoms of the cyclam ligands and one S atom of the [SbS_4_]^3–^ anions within a square-pyramidal coordination (Fig. 3 ▸). All of the cyclam ligands adopt the *trans*-IV(*R*,*S*,*S*,*R*) configuration. One of the cyclam ligands and both perchlorate anions are disordered over two orientations and were refined using a split model (see *Refinement*). The Zn—N and Zn—S bond lengths (Table 1 ▸) vary slightly between the four independent complex cations but correspond to values comparable to those in other Zn(cyclam)^2+^ cations. From the bond angles it can be seen that the square-pyramidal coordination geometry is slightly distorted (see supporting information). The Zn^2+^ cations are shifted out of the N_4_ plane of the cyclam ligands by 0.5068 (4) Å (Zn1), 0.4758 (4) Å (Zn11), 0.4685 (5) and 0.541 Å (Zn21) and 0.4879 (4) Å (Zn31), similar to what is usually observed in Zn compounds containing cyclam ligands. In this context, it is noted that the asymmetric coordination of the Zn^2+^ cations in compounds where Zn(cyclam)^2+^ cations are sixfold coordinated frequently leads to a disorder of the Zn^2+^ cation that is either located above and below the N_4_ plane, as is the case, for example, in [Zn(cyclam)]_3_[SbS_4_]_2_·8H_2_O (CSD refcode GALPUI; Danker *et al.*, 2021 ▸), Zn(cyclam)(methyl­carbon­ato)(perchlorate) (CUZHUA; Kato & Ito, 1985 ▸), Zn(NCS)_2_(cyclam) (DITZIP; Ito *et al.*, 1984 ▸) and Zn*X*_2_(cyclam) with *X* = Cl, Br, I (HEGNEM, HEGNOW and VUSDUI10; Porai-Koshits *et al.*, 1994 ▸).

Each pair of Zn(cyclam)^2+^ cations is linked *via* the S atoms of the [SbS_4_]^3–^ anions into [Zn_2_(cyclam)_2_SbS_4_]^+^ cations (Fig. 3 ▸) and charge balance is achieved by additional perchlorate anions, both of them disordered in two different orientations. The Sb—S bond lengths to the two S atoms that are involved in metal coordination are significantly longer than that to the other two S atoms (Table 1 ▸). The S—Sb—S angles show that the tetra­hedra are slightly distorted (see supporting information).

## 3. Supra­molecular features

**Figure 4:**
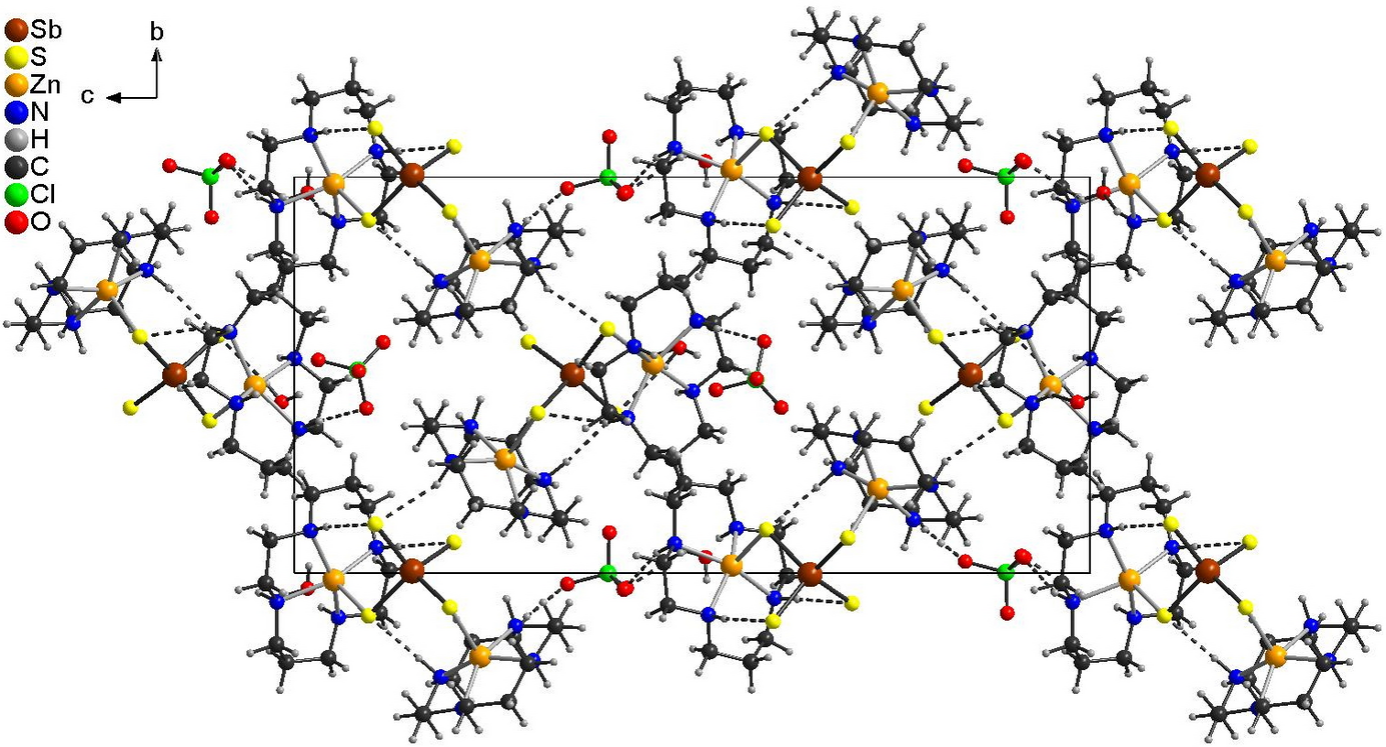
The crystal structure of (I) viewed along the crystallographic *a*-axis direction with N—H⋯S and N—H⋯O hydrogen bonds shown as dashed lines. The disorder is omitted for clarity.

The crystal structure of the title compound is dominated by numerous N—H⋯O and N—H⋯S hydrogen bonds (Table 2 ▸) but several of them show angles that are far from linearity and some of them are close to the sum of the van der Waals radii. Therefore, in Table 2 ▸ and Fig. 4 ▸ only those with angles smaller than 140° and, for example, S⋯H distances shorter than 2.6 Å are considered. In this case, the N—H hydrogen atoms of the cyclam ligands are connected *via* N—H⋯S hydrogen bonds to the [SbS_4_]^3–^ anions. Some of them are close to linear with relatively short S⋯H distances, which indicates that these correspond to strong inter­actions (Fig. 4 ▸ and Table 2 ▸). There is also N—H⋯O hydrogen bonding to the perchlorate anions and one of the water mol­ecules (Fig. 4 ▸ and Table 2 ▸). The other water mol­ecule might also be involved in hydrogen bonding but the distances and angles indicate that these are only weak inter­actions. In this context, it is noted that the O—H hydrogen atoms were clearly located in difference maps (see *Refinement*). There is a very large number of contacts between the C—H hydrogen atoms of the cyclam ligands and the O and S atoms of the perchlorate and tetra­thio­anti­monate anions as well the water O atoms, but from the values of the distances and angles they should not correspond to strong inter­actions.

## 4. Database survey

A search for structures of zinc–cylam complexes in the Cambridge Structural database (CSD version 5.42, last update November 2021; Groom *et al.*, 2016 ▸) leads to 37 hits, of which 34 correspond to a sixfold and three to a twofold coordination. In only two of them is the Zn(cyclam) cation coordinated to a sulfur atom and both of these show a square pyramidal coordination (ICUFES and ICUFIW; Notni *et al.*, 2006 ▸). None of these structures contains thio­anti­monate anions. However, as mentioned in the *Chemical context*, one compound with the composition [(Zn-cyclam)_3_(SbS_4_)_2_]·8H_2_O has been reported and in this structure the Zn cations are fivefold coordinated but disordered over both N_4_ planes of the cyclam ligand (Danker *et al.*, 2021 ▸). Three Zn compounds with thio­anti­monate anions are also known, *viz*. Zn(tri­ethyl­eneteramine)Sb_4_S_7_ (CODQOC; Lühmann, *et al.*, 2008 ▸), Zn(tris­(2-amino­eth­yl)amineSb_4_S_7_ (JALZIG; Schaefer *et al.*, 2004*a* ▸) and Zn(tris­(2-amino­eth­yl)amine)_2_Sb_4_S_8_ (PANCUD; Schaefer *et al.*, 2004*b* ▸).

## 5. Additional investigations

**Figure 5:**
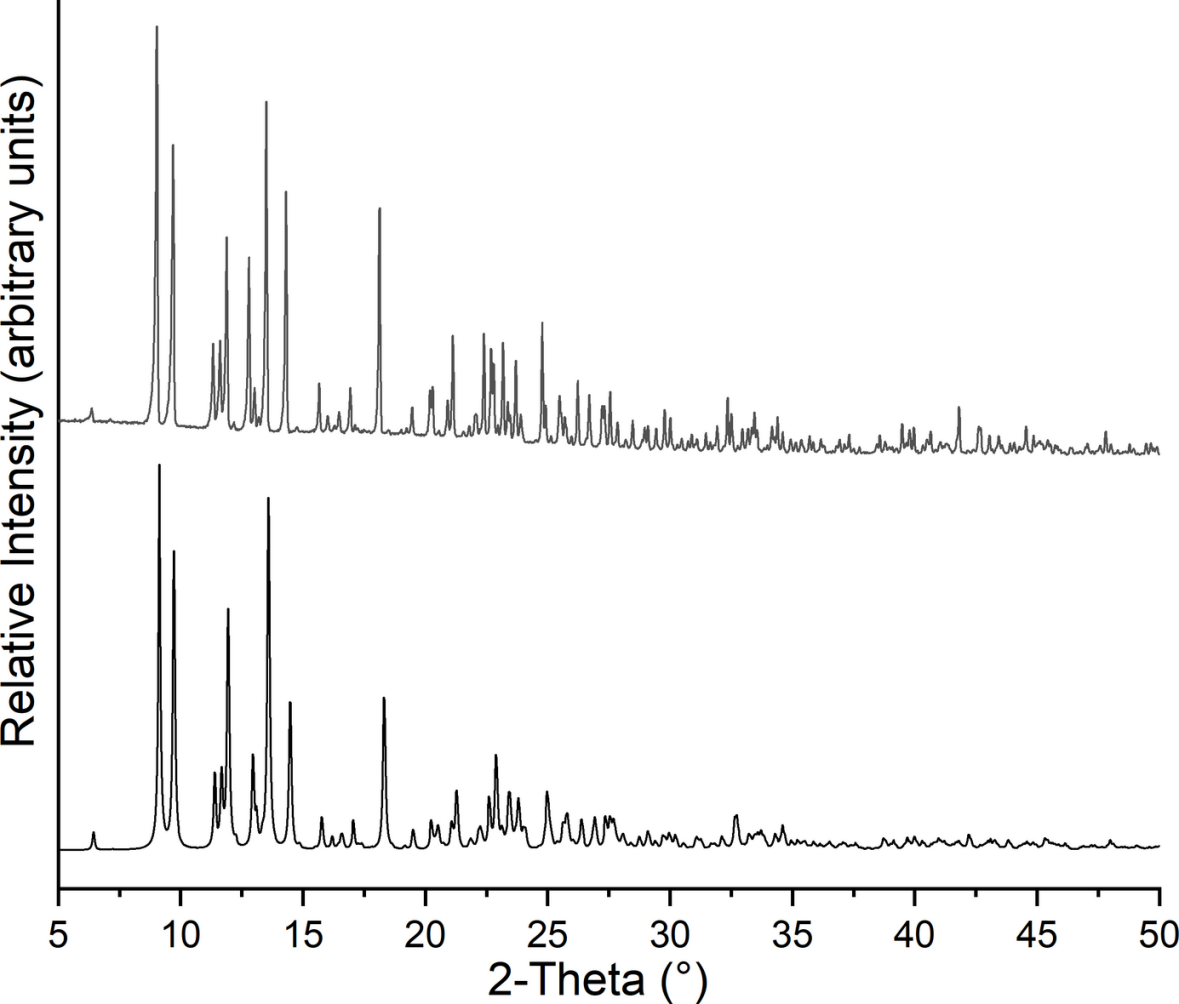
Experimental (top) and calculated (bottom) powder patterns for (I).

**Figure 6:**
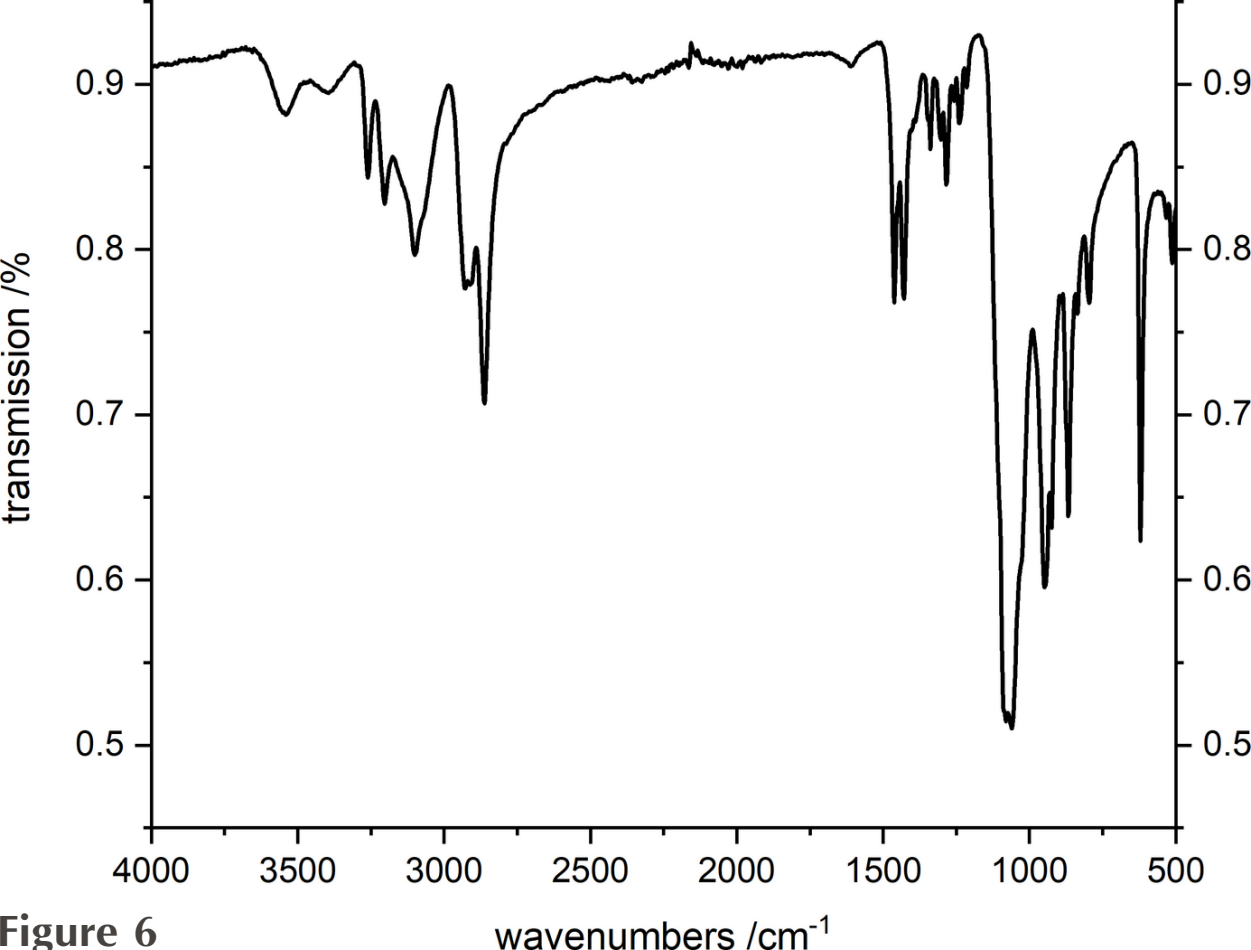
IR spectrum of (I).

**Figure 7:**
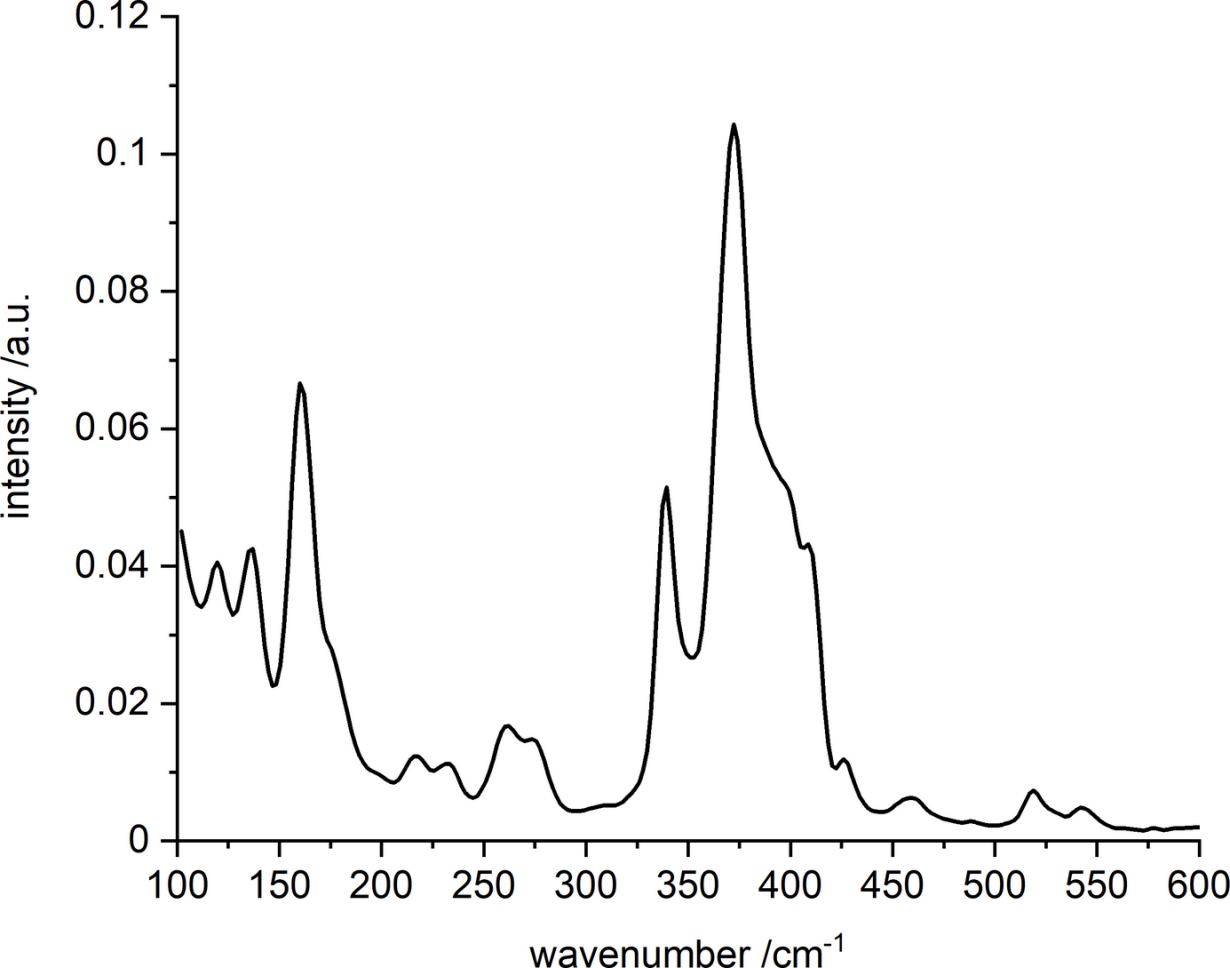
Raman spectrum of (I).

Comparison of the experimental powder pattern of (I) with that calculated from single-crystal data proves that a pure phase was obtained (Fig. 5 ▸). The IR spectrum (Fig. 6 ▸) shows the O—H stretching modes at 3550 and 3400 cm^−1^ and the N—H related bands are at 3260, 3208 and 3103 cm^−1^. The bands of the CH_2_ groups are found at 2930, 2910, and 2862 cm^−1^. The absorptions at 1461 and 1430 cm^−1^ cannot be assigned unambiguously because C—C, C—N stretching and CH deformation modes are located in this region. The very strong absorptions at 1083 and 1060 cm^−1^ are related to the ClO_4_^−^ anion (Zapata & García-Ruiz, 2018 ▸; Hillebrecht *et al.*, 1994 ▸). However, in this region C—N stretching and the CH_2_ deformation vibrations also occur, which overlap with the bands of the perchlorate ions. The *trans* and *cis* isomers of coordinated cyclam show different absorptions between 790 and 910 cm^−1^, *i.e.* only three bands occur for the *trans* isomer and six for the *cis* configuration (Poon, 1971 ▸). As expected, the IR spectrum contains only three absorptions at 866, 835, and 794 cm^−1^. The band at 621 cm^−1^ is caused by the deformation vibration of the perchlorate anion. In the Raman spectrum (Fig. 7 ▸) four resonances can be expected for the ideal [SbS_4_]^3–^ anion, which are located at 388, 366, 178, and 156 cm^−1^ in Na_3_SbS_4_ (Mikenda & Preisinger, 1980 ▸). Because the ideal *T*_d_ symmetry of the two independent thio­anti­monate(V) anions is significantly reduced, a more complex Raman spectrum is observed. The intense resonance at 372 cm^−1^ has shoulders at higher energies at 398 and 406 cm^−1^. The occurrence of the three bands is most probably caused by the differing Sb—S bond lengths. The deformation resonances of the anions are located at 161 and 175 (shoulder) cm^−1^. The relatively weak band at 338 cm^−1^ is most probably caused by the cyclam ligand (Danker *et al.*, 2021 ▸). According to this reference, the Zn—S related resonance is weak and occurs at 262 cm^−1^.

## 6. Synthesis and crystallization

**Synthesis of Na_3_SbS_4_·9H_2_O**

Na_3_SbS_4_·9H_2_O was synthesized by adding 16.6 g (0.213 mol) of Na_2_S·*x*H_2_O (technical grade, purchased from Acros Organics) to 58 ml of demineralized water. This solution was heated to 323 K for 1 h. Afterwards 19.6 g (0.058 mol) of Sb_2_S_3_ (98%, purchased from Alfa Aesar) and 3.69 g (0.115 mol) of sulfur (min. 99%, purchased from Alfa Aesar), were added and the reaction mixture was heated to 343 K for 6 h. The reaction mixture was filtered and the filtrate was stored overnight, leading to the formation of slightly yellow crystals, which were filtered off, washed with small amounts of water and dried under vacuum (yield about 30% based on Sb_2_S_3_).

**Synthesis of the title compound**

16 mg (0.044 mmol) of Zn(ClO_4_)_2_·6H_2_O (purchased from Alfa Aesar) and 16 mg (0.08 mmol) of cyclam (purchased from Strem Chemicals) were dissolved in 2 ml of aceto­nitrile (purchased from Merck). To this solution, a solution of 50 mg (0.14 mmol) of Na_3_SbS_4_·9H_2_O dissolved in 1 ml of H_2_O was added. Within 3 d, a few colorless crystals of the title compound were obtained.

**Experimental details**

The PXRD measurements were performed with Cu *K*α_1_ radiation (λ = 1.540598 Å) using a Stoe Transmission Powder Diffraction System (STADI P) equipped with a MYTHEN 1K detector and a Johansson-type Ge(111) monochromator. The IR spectra were measured using an ATI Mattson Genesis Series FTIR Spectrometer, control software: *WINFIRST*, from ATI Mattson. The Raman spectra were recorded at room temperature on a Bruker RAM II FT-Raman spectrometer using a liquid nitro­gen cooled, highly sensitive Ge detector at 1064 nm radiation and with 3 cm^−1^ resolution.

## 7. Refinement

Crystal data, data collection and structure refinement details are summarized in Table 3 ▸. All non-hydrogen atoms were refined anisotropically. The C- and N-bound H atoms were positioned with idealized geometry and were refined isotrop­ically with *U*_iso_(H) = 1.2*U*_eq_(C,N) using a riding model. The O-bound H atoms were located in difference maps, their bond lengths were set to ideal values and they were refined isotropically with *U*_iso_(H) = 1.5*U*_eq_(O) using a riding model. Both crystallographically independent perchlorate anions are disordered over two orientations and were refined using a split model with restraints for the geometry and the components of the anisotropic displacement parameters. Even in this case, relatively large displacement parameters are observed, indicating that more than two orientations are probably involved. Complete disorder is also observed for one of the four crystallographically independent cyclam ligands, which also was refined using a split model and restraints, but even in this case some of the atoms show relatively high components of their anisotropic displacement parameters. The Zn cation coordinated by this ligand exhibits anisotropic displacement parameters that are slightly higher than those of the other Zn cations and close to this cation the highest maximum in the difference map is observed, which indicates that the position of this cation is also influenced by the disorder. The possible Zn disorder cannot be resolved. It is noted that the ratio between the site occupation factors (sof) of the disordered perchlorate anions of 0.8:0.2 is slightly different from that observed in the cyclam ligand. However, they need not necessarily depend on each other and precise determination of the sof’s is difficult to achieve. In the end we selected ratios where the best reliability factors were observed. It is also noted that the sof of the water mol­ecules is identical to the occupancy of the major disorder component of the perchlor­ate anions, but they do not necessarily depend on each other: it is possible that some amount of water was lost on storage and that the water positions were fully occupied in freshly prepared crystals. The structure was refined as a racemic twin, leading to a BASF parameter of 0.408 (5). It was also attempted to refine the structure in the centrosymmetric space group *P*2_1_/*c* but in this case much disorder is observed and no reasonable structural model can be found. In this context it is noted that *checkCIF* does not suggest higher symmetry.

## Supplementary Material

Crystal structure: contains datablock(s) I. DOI: 10.1107/S2056989024009356/hb8108sup1.cif

Structure factors: contains datablock(s) I. DOI: 10.1107/S2056989024009356/hb8108Isup2.hkl

CCDC reference: 2385974

Additional supporting information:  crystallographic information; 3D view; checkCIF report

## Figures and Tables

**Figure 2:**
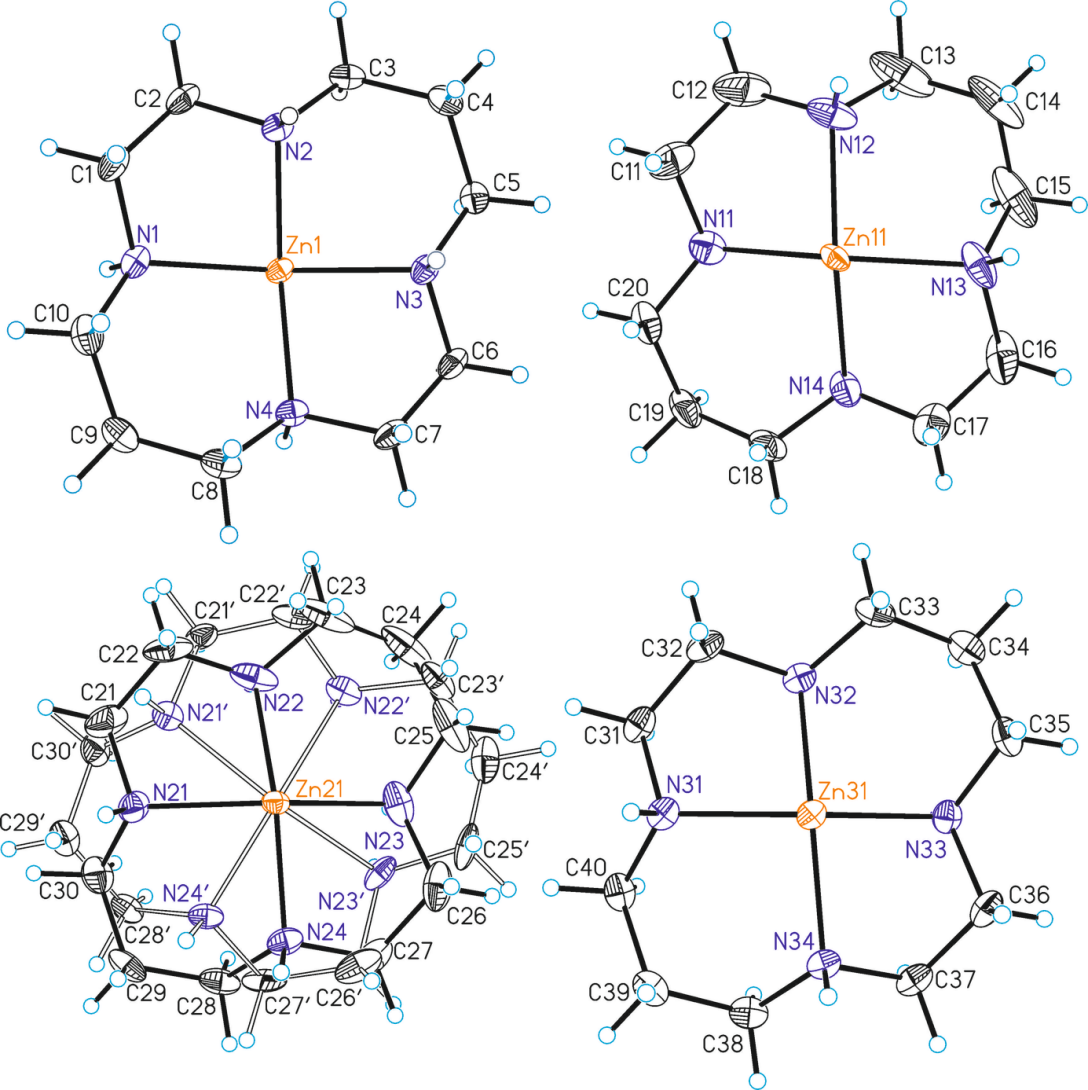
The Zn(cyclam) complexes in (I) with labeling and displacement ellipsoids drawn at the 50% probability level. The disorder of the cyclam ligands is shown with full and open bonds.

**Table 1:** Selected bond lengths (Å)

Sb1—S1	2.3409 (7)	Sb11—S13	2.3030 (7)
Sb1—S2	2.3593 (7)	Sb11—S14	2.3100 (7)
Sb1—S3	2.3090 (7)	S11—Zn21	2.3806 (7)
Sb1—S4	2.3168 (7)	S12—Zn31	2.3870 (8)
S1—Zn1	2.4024 (7)	Zn21—N21	2.166 (4)
S2—Zn11	2.3694 (8)	Zn21—N22	2.115 (4)
Zn1—N1	2.137 (2)	Zn21—N23	2.080 (4)
Zn1—N2	2.118 (2)	Zn21—N24	2.133 (3)
Zn1—N3	2.144 (2)	Zn21—N21′	2.143 (9)
Zn1—N4	2.116 (2)	Zn21—N22′	2.239 (9)
Zn11—N11	2.107 (3)	Zn21—N23′	2.144 (8)
Zn11—N12	2.124 (3)	Zn21—N24′	2.054 (8)
Zn11—N13	2.135 (3)	Zn31—N31	2.114 (3)
Zn11—N14	2.134 (3)	Zn31—N32	2.141 (2)
Sb11—S11	2.3424 (7)	Zn31—N33	2.121 (3)
Sb11—S12	2.3572 (8)	Zn31—N34	2.137 (2)

**Table 2:** Hydrogen-bond geometry (Å, °)

*D*—H⋯*A*	*D*—H	H⋯*A*	*D*⋯*A*	*D*—H⋯*A*
N4—H4⋯S2	1.00	2.30	3.289 (2)	172
N11—H11⋯O42	1.00	2.00	2.948 (5)	157
N12—H12⋯S4	1.00	2.40	3.404 (3)	178
N13—H13⋯S3	1.00	2.46	3.444 (3)	169
N14—H14⋯O24	1.00	2.17	3.135 (6)	163
N14—H14⋯O31	1.00	2.28	3.115 (19)	140
N14—H14⋯O33	1.00	2.47	3.41 (4)	156
N22—H22⋯O3	1.00	2.16	3.158 (6)	175
N23—H23⋯O41	1.00	2.35	3.235 (6)	147
N24—H24⋯S12	1.00	2.56	3.483 (3)	154
N22′—H22′⋯O12	1.00	2.29	3.27 (3)	166
N31—H31⋯S14	1.00	2.36	3.355 (3)	174
N33—H33⋯S3^i^	1.00	2.53	3.419 (3)	148
N34—H34⋯S13	1.00	2.58	3.532 (2)	159
O41—H41*A*⋯S14^ii^	0.82	2.48	3.256 (4)	158

Symmetry codes: (i) 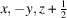; (ii) 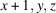.

**Table 3:** Experimental details

Crystal data	
Chemical formula	[Zn_2_(SbS_4_)(C_10_H_24_N_4_)_2_]ClO_4_·0.8H_2_O
*M*_r_	895.25
Crystal system, space group	Monoclinic, *Pc*
Temperature (K)	100
*a*, *b*, *c* (Å)	9.11238 (7), 13.67434 (10), 27.49214 (18)
β (°)	92.9206 (6)
*V* (Å^3^)	3421.23 (4)
*Z*	4
Radiation type	Mo *K*α
μ (mm^−1^)	2.54
Crystal size (mm)	0.2 × 0.2 × 0.15

Data collection	
Diffractometer	XtaLAB Synergy, Dualflex, HyPix
Absorption correction	Multi-scan (*CrysAlis PRO*; Rigaku OD, 2021 ▸)
*T*_min_, *T*_max_	0.856, 1.000
No. of measured, independent and observed [*I* > 2σ(*I*)] reflections	112309, 24308, 23520
*R*_int_	0.021
(sin θ/λ)_max_ (Å^−1^)	0.783

Refinement	
*R*[*F*^2^ > 2σ(*F*^2^)], *wR*(*F*^2^), *S*	0.023, 0.058, 1.03
No. of reflections	24308
No. of parameters	956
No. of restraints	314
H-atom treatment	H-atom parameters constrained
Δρ_max_, Δρ_min_ (e Å^−3^)	2.16, −0.58
Absolute structure	Refined as an inversion twin
Absolute structure parameter	0.408 (5)

Computer programs: *CrysAlis PRO* (Rigaku OD, 2021 ▸), *SHELXT2014/5* (Sheldrick, 2015*a* ▸), *SHELXL2016/6* (Sheldrick, 2015*b* ▸), *DIAMOND* (Brandenburg, 1999 ▸) and *publCIF* (Westrip, 2010 ▸).

## supplementary crystallographic information

### (µ-Tetrathioantimonato-κ2S:S')bis[(1,4,8,11-tetraazacyclotetradecane-κ4N)zinc(II)] perchlorate 0.8-hydrate Crystal data

**Table:**

[Zn_2_(SbS_4_)(C_10_H_24_N_4_)_2_]ClO_4_·0.8H_2_O	*F*(000) = 1824
*M**_r_* = 895.25	*D*_x_ = 1.738 Mg m^−^^3^
Monoclinic, *P**c*	Mo *K*α radiation, λ = 0.71073 Å
*a* = 9.11238 (7) Å	Cell parameters from 77740 reflections
*b* = 13.67434 (10) Å	θ = 2.7–33.8°
*c* = 27.49214 (18) Å	µ = 2.54 mm^−^^1^
β = 92.9206 (6)°	*T* = 100 K
*V* = 3421.23 (4) Å^3^	Block, colourless
*Z* = 4	0.2 × 0.2 × 0.15 mm

### (µ-Tetrathioantimonato-κ2S:S')bis[(1,4,8,11-tetraazacyclotetradecane-κ4N)zinc(II)] perchlorate 0.8-hydrate Data collection

**Table:**

XtaLAB Synergy, Dualflex, HyPix diffractometer	24308 independent reflections
Radiation source: micro-focus sealed X-ray tube, PhotonJet (Mo) X-ray Source	23520 reflections with *I* > 2σ(*I*)
Mirror monochromator	*R*_int_ = 0.021
Detector resolution: 10.0000 pixels mm^-1^	θ_max_ = 33.8°, θ_min_ = 2.1°
ω scans	*h* = −14→14
Absorption correction: multi-scan (CrysAlisPro; Rigaku OD, 2021)	*k* = −21→20
*T*_min_ = 0.856, *T*_max_ = 1.000	*l* = −42→41
112309 measured reflections	

### (µ-Tetrathioantimonato-κ2S:S')bis[(1,4,8,11-tetraazacyclotetradecane-κ4N)zinc(II)] perchlorate 0.8-hydrate Refinement

**Table:**

Refinement on *F*^2^	Hydrogen site location: mixed
Least-squares matrix: full	H-atom parameters constrained
*R*[*F*^2^ > 2σ(*F*^2^)] = 0.023	*w* = 1/[σ^2^(*F*_o_^2^) + (0.0348*P*)^2^ + 1.8205*P*] where *P* = (*F*_o_^2^ + 2*F*_c_^2^)/3
*wR*(*F*^2^) = 0.058	(Δ/σ)_max_ = 0.002
*S* = 1.02	Δρ_max_ = 2.16 e Å^−^^3^
24308 reflections	Δρ_min_ = −0.58 e Å^−^^3^
956 parameters	Absolute structure: Refined as an inversion twin
314 restraints	Absolute structure parameter: 0.408 (5)
Primary atom site location: dual	

### (µ-Tetrathioantimonato-κ2S:S')bis[(1,4,8,11-tetraazacyclotetradecane-κ4N)zinc(II)] perchlorate 0.8-hydrate Special details

**Table:**

Geometry. All esds (except the esd in the dihedral angle between two l.s. planes) are estimated using the full covariance matrix. The cell esds are taken into account individually in the estimation of esds in distances, angles and torsion angles; correlations between esds in cell parameters are only used when they are defined by crystal symmetry. An approximate (isotropic) treatment of cell esds is used for estimating esds involving l.s. planes.
Refinement. Refined as a 2-component inversion twin.

### (µ-Tetrathioantimonato-κ2S:S')bis[(1,4,8,11-tetraazacyclotetradecane-κ4N)zinc(II)] perchlorate 0.8-hydrate Fractional atomic coordinates and isotropic or equivalent isotropic displacement parameters (Å^2^)

**Table:**

	*x*	*y*	*z*	*U*_iso_*/*U*_eq_	Occ. (<1)
Sb1	0.48949 (2)	−0.00451 (2)	0.35226 (2)	0.01278 (3)	
S1	0.64411 (8)	0.08789 (5)	0.30469 (3)	0.01815 (12)	
S2	0.39294 (8)	0.10867 (5)	0.40697 (2)	0.01748 (12)	
S3	0.61087 (8)	−0.12292 (5)	0.39871 (3)	0.01883 (12)	
S4	0.31484 (8)	−0.07701 (5)	0.29972 (3)	0.01926 (12)	
Zn1	0.49094 (3)	0.21190 (2)	0.26659 (2)	0.01471 (6)	
N1	0.3300 (3)	0.13456 (18)	0.22234 (9)	0.0186 (4)	
H1	0.336318	0.065002	0.233481	0.022*	
C1	0.3798 (4)	0.1343 (2)	0.17181 (11)	0.0219 (6)	
H1A	0.340248	0.192494	0.154180	0.026*	
H1B	0.341949	0.075270	0.154527	0.026*	
C2	0.5474 (4)	0.1354 (2)	0.17217 (11)	0.0222 (6)	
H2A	0.587943	0.074994	0.187533	0.027*	
H2B	0.580474	0.139093	0.138473	0.027*	
N2	0.5981 (3)	0.22173 (17)	0.20017 (9)	0.0177 (4)	
H2	0.560807	0.281365	0.182492	0.021*	
C3	0.7598 (3)	0.2295 (2)	0.20650 (11)	0.0203 (5)	
H3A	0.803114	0.225123	0.174283	0.024*	
H3B	0.797998	0.174231	0.226636	0.024*	
C4	0.8062 (3)	0.3256 (2)	0.23085 (11)	0.0216 (5)	
H4A	0.760125	0.379903	0.211775	0.026*	
H4B	0.913962	0.332392	0.229069	0.026*	
C5	0.7678 (3)	0.3378 (2)	0.28401 (11)	0.0201 (5)	
H5A	0.808934	0.282277	0.303354	0.024*	
H5B	0.813173	0.398696	0.297215	0.024*	
N3	0.6070 (3)	0.34206 (18)	0.28886 (8)	0.0170 (4)	
H3	0.567494	0.397397	0.268344	0.020*	
C6	0.5625 (4)	0.3578 (2)	0.33895 (10)	0.0200 (5)	
H6A	0.600329	0.421381	0.351418	0.024*	
H6B	0.602719	0.305329	0.360576	0.024*	
C7	0.3952 (4)	0.3568 (2)	0.33779 (11)	0.0210 (6)	
H7A	0.361538	0.363831	0.371282	0.025*	
H7B	0.356063	0.412553	0.318159	0.025*	
N4	0.3391 (3)	0.26367 (17)	0.31631 (9)	0.0174 (4)	
H4	0.345431	0.215790	0.343828	0.021*	
C8	0.1801 (3)	0.2687 (2)	0.30146 (11)	0.0217 (5)	
H8A	0.162617	0.324287	0.278811	0.026*	
H8B	0.122903	0.280543	0.330562	0.026*	
C9	0.1272 (3)	0.1744 (2)	0.27670 (12)	0.0232 (6)	
H9A	0.018615	0.172132	0.276787	0.028*	
H9B	0.165711	0.118292	0.296214	0.028*	
C10	0.1727 (3)	0.1619 (3)	0.22442 (12)	0.0246 (6)	
H10A	0.111148	0.110624	0.208184	0.030*	
H10B	0.154970	0.223905	0.206459	0.030*	
Zn11	0.22128 (4)	0.01830 (3)	0.45074 (2)	0.01828 (6)	
N11	0.0381 (3)	0.1123 (2)	0.44475 (11)	0.0282 (5)	
H11	−0.039249	0.087372	0.466100	0.034*	
C11	−0.0142 (4)	0.1011 (4)	0.39304 (15)	0.0397 (9)	
H11A	0.057343	0.130945	0.371561	0.048*	
H11B	−0.109648	0.134859	0.387364	0.048*	
C12	−0.0317 (5)	−0.0072 (4)	0.3813 (2)	0.0496 (13)	
H12A	−0.117986	−0.033123	0.397511	0.060*	
H12B	−0.050147	−0.015229	0.345678	0.060*	
N12	0.0994 (4)	−0.0640 (2)	0.39710 (12)	0.0355 (7)	
H12	0.161917	−0.066223	0.368267	0.043*	
C13	0.0662 (6)	−0.1670 (4)	0.4083 (2)	0.0602 (16)	
H13A	0.023630	−0.198838	0.378436	0.072*	
H13B	−0.008748	−0.168835	0.433069	0.072*	
C14	0.1990 (6)	−0.2247 (3)	0.4268 (2)	0.0568 (15)	
H14A	0.279811	−0.213645	0.404692	0.068*	
H14B	0.174375	−0.295170	0.425661	0.068*	
C15	0.2529 (6)	−0.1983 (3)	0.4784 (2)	0.0553 (14)	
H15A	0.167496	−0.192075	0.499067	0.066*	
H15B	0.316065	−0.251708	0.491926	0.066*	
N13	0.3367 (4)	−0.1060 (2)	0.48003 (11)	0.0339 (7)	
H13	0.423441	−0.116132	0.459756	0.041*	
C16	0.3963 (5)	−0.0824 (3)	0.53088 (13)	0.0419 (10)	
H16A	0.328096	−0.106903	0.554986	0.050*	
H16B	0.492550	−0.114870	0.537123	0.050*	
C17	0.4139 (5)	0.0265 (4)	0.53614 (13)	0.0369 (9)	
H17A	0.443925	0.043150	0.570254	0.044*	
H17B	0.490822	0.050143	0.514852	0.044*	
N14	0.2726 (3)	0.0731 (2)	0.52227 (10)	0.0274 (5)	
H14	0.196565	0.050094	0.544620	0.033*	
C18	0.2776 (4)	0.1806 (3)	0.52279 (13)	0.0301 (7)	
H18A	0.348261	0.203340	0.499049	0.036*	
H18B	0.313328	0.203111	0.555514	0.036*	
C19	0.1289 (4)	0.2259 (2)	0.51035 (13)	0.0317 (7)	
H19A	0.134568	0.296525	0.518180	0.038*	
H19B	0.056638	0.196318	0.531667	0.038*	
C20	0.0706 (4)	0.2150 (2)	0.45749 (14)	0.0303 (7)	
H20A	−0.019921	0.254449	0.452345	0.036*	
H20B	0.144527	0.240725	0.435606	0.036*	
Sb11	0.45265 (2)	0.50033 (2)	0.65050 (2)	0.01381 (4)	
S11	0.58221 (8)	0.61580 (5)	0.60663 (2)	0.01812 (12)	
S12	0.62781 (8)	0.40364 (5)	0.69441 (3)	0.02223 (13)	
S13	0.31876 (8)	0.58313 (5)	0.70617 (2)	0.01719 (12)	
S14	0.32052 (9)	0.40053 (5)	0.59646 (3)	0.02177 (13)	
Zn21	0.72146 (4)	0.52864 (2)	0.54987 (2)	0.01663 (6)	
N21	0.5613 (4)	0.4593 (3)	0.50014 (13)	0.0243 (7)	0.72
H21	0.468063	0.456420	0.517748	0.029*	0.72
C21	0.5320 (8)	0.5260 (5)	0.45876 (19)	0.0357 (13)	0.72
H21A	0.428346	0.518976	0.446648	0.043*	0.72
H21B	0.595477	0.508149	0.431968	0.043*	0.72
C22	0.5609 (7)	0.6309 (4)	0.47346 (17)	0.0365 (12)	0.72
H22A	0.549114	0.674327	0.444716	0.044*	0.72
H22B	0.490856	0.651855	0.497772	0.044*	0.72
N22	0.7124 (5)	0.6360 (3)	0.49444 (14)	0.0324 (9)	0.72
H22	0.780914	0.616550	0.468866	0.039*	0.72
C23	0.7569 (11)	0.7339 (5)	0.5135 (3)	0.0463 (19)	0.72
H23A	0.692503	0.752723	0.539835	0.056*	0.72
H23B	0.744135	0.782819	0.487062	0.056*	0.72
C24	0.9149 (8)	0.7342 (4)	0.5328 (3)	0.0482 (15)	0.72
H24A	0.945731	0.803027	0.537883	0.058*	0.72
H24B	0.976038	0.706426	0.507481	0.058*	0.72
C25	0.9489 (8)	0.6790 (5)	0.5798 (3)	0.0430 (14)	0.72
H25A	1.050746	0.693768	0.591736	0.052*	0.72
H25B	0.881819	0.701382	0.604693	0.052*	0.72
N23	0.9325 (4)	0.5717 (3)	0.57311 (15)	0.0307 (8)	0.72
H23	1.002006	0.549972	0.548257	0.037*	0.72
C26	0.9615 (6)	0.5130 (5)	0.6177 (2)	0.0346 (12)	0.72
H26A	0.895488	0.533773	0.643320	0.042*	0.72
H26B	1.064424	0.522148	0.630200	0.042*	0.72
C27	0.9338 (12)	0.4057 (8)	0.6045 (4)	0.035 (2)	0.72
H27A	1.007688	0.383842	0.581641	0.042*	0.72
H27B	0.945047	0.365141	0.634235	0.042*	0.72
N24	0.7844 (4)	0.3917 (3)	0.58161 (12)	0.0221 (6)	0.72
H24	0.717309	0.379446	0.608649	0.027*	0.72
C28	0.7764 (6)	0.3039 (3)	0.5498 (2)	0.0285 (9)	0.72
H28A	0.802659	0.245452	0.569649	0.034*	0.72
H28B	0.849563	0.310389	0.524609	0.034*	0.72
C29	0.6259 (5)	0.2887 (3)	0.52506 (19)	0.0282 (9)	0.72
H29A	0.551004	0.295581	0.549695	0.034*	0.72
H29B	0.619845	0.220914	0.512415	0.034*	0.72
C30	0.5883 (6)	0.3588 (4)	0.48334 (19)	0.0289 (9)	0.72
H30A	0.670171	0.359623	0.461029	0.035*	0.72
H30B	0.499541	0.334780	0.464766	0.035*	0.72
N21'	0.5879 (10)	0.5630 (6)	0.4857 (3)	0.0196 (15)	0.28
H21'	0.490707	0.582483	0.498030	0.023*	0.28
C21'	0.6444 (12)	0.6524 (8)	0.4632 (4)	0.0238 (19)	0.28
H21C	0.561363	0.689060	0.447274	0.029*	0.28
H21D	0.712228	0.633953	0.437698	0.029*	0.28
C22'	0.7235 (17)	0.7169 (11)	0.4999 (6)	0.025 (3)	0.28
H22C	0.765621	0.773990	0.483403	0.029*	0.28
H22D	0.653969	0.741190	0.523647	0.029*	0.28
N22'	0.8390 (9)	0.6618 (6)	0.5248 (3)	0.0220 (16)	0.28
H22'	0.909830	0.640864	0.500195	0.026*	0.28
C23'	0.9222 (16)	0.7182 (10)	0.5637 (5)	0.029 (2)	0.28
H23C	0.853360	0.741764	0.587848	0.035*	0.28
H23D	0.968408	0.776008	0.549096	0.035*	0.28
C24'	1.0403 (14)	0.6551 (10)	0.5892 (5)	0.035 (2)	0.28
H24C	1.097257	0.697123	0.612596	0.042*	0.28
H24D	1.108414	0.633556	0.564360	0.042*	0.28
C25'	0.9916 (12)	0.5648 (11)	0.6164 (4)	0.030 (2)	0.28
H25C	1.076472	0.537189	0.635690	0.037*	0.28
H25D	0.915841	0.583501	0.639277	0.037*	0.28
N23'	0.9325 (9)	0.4919 (7)	0.5829 (3)	0.0227 (17)	0.28
H23'	1.003186	0.481096	0.556799	0.027*	0.28
C26'	0.900 (3)	0.395 (2)	0.6066 (10)	0.034 (4)	0.28
H26C	0.992822	0.362183	0.617392	0.040*	0.28
H26D	0.841507	0.405653	0.635555	0.040*	0.28
C27'	0.8145 (13)	0.3305 (9)	0.5703 (5)	0.029 (2)	0.28
H27C	0.880332	0.309603	0.544727	0.035*	0.28
H27D	0.781429	0.271038	0.587193	0.035*	0.28
N24'	0.6863 (9)	0.3803 (6)	0.5475 (3)	0.0199 (15)	0.28
H24'	0.603400	0.367618	0.569099	0.024*	0.28
C28'	0.6412 (14)	0.3360 (8)	0.4995 (4)	0.024 (2)	0.28
H28C	0.613780	0.266763	0.504469	0.028*	0.28
H28D	0.725945	0.337019	0.478318	0.028*	0.28
C29'	0.5114 (13)	0.3895 (9)	0.4735 (4)	0.025 (2)	0.28
H29C	0.466844	0.346148	0.447986	0.030*	0.28
H29D	0.435954	0.402462	0.497390	0.030*	0.28
C30'	0.5536 (15)	0.4853 (9)	0.4504 (4)	0.025 (2)	0.28
H30C	0.640324	0.474287	0.430772	0.030*	0.28
H30D	0.471730	0.507193	0.427927	0.030*	0.28
Zn31	0.48590 (4)	0.28608 (2)	0.73574 (2)	0.01639 (6)	
N31	0.3173 (3)	0.23507 (18)	0.68650 (9)	0.0192 (4)	
H31	0.318186	0.280393	0.657976	0.023*	
C31	0.3626 (4)	0.1390 (2)	0.66769 (12)	0.0225 (6)	
H31A	0.316631	0.128309	0.634709	0.027*	
H31B	0.329365	0.086252	0.689234	0.027*	
C32	0.5304 (4)	0.1359 (2)	0.66563 (11)	0.0226 (6)	
H32A	0.562309	0.071332	0.653822	0.027*	
H32B	0.564176	0.186896	0.643205	0.027*	
N32	0.5924 (3)	0.15367 (18)	0.71550 (9)	0.0191 (4)	
H32	0.559852	0.099744	0.737038	0.023*	
C33	0.7548 (3)	0.1589 (2)	0.71938 (12)	0.0243 (6)	
H33A	0.788264	0.213519	0.699105	0.029*	
H33B	0.796139	0.097485	0.706758	0.029*	
C34	0.8115 (4)	0.1741 (2)	0.77188 (12)	0.0245 (6)	
H34A	0.919735	0.167183	0.773190	0.029*	
H34B	0.772201	0.120807	0.791864	0.029*	
C35	0.7741 (3)	0.2713 (2)	0.79542 (12)	0.0241 (6)	
H35A	0.828208	0.276746	0.827451	0.029*	
H35B	0.806142	0.325651	0.774654	0.029*	
N33	0.6148 (3)	0.28027 (18)	0.80214 (9)	0.0198 (4)	
H33	0.582528	0.222678	0.821298	0.024*	
C36	0.5738 (4)	0.3706 (2)	0.82780 (12)	0.0248 (6)	
H36A	0.608745	0.428847	0.810427	0.030*	
H36B	0.618666	0.370949	0.861363	0.030*	
C37	0.4070 (4)	0.3719 (2)	0.82894 (11)	0.0230 (6)	
H37A	0.373145	0.314560	0.847261	0.028*	
H37B	0.374945	0.431815	0.845639	0.028*	
N34	0.3415 (3)	0.36956 (17)	0.77840 (9)	0.0184 (4)	
H34	0.347013	0.438194	0.766064	0.022*	
C38	0.1826 (3)	0.3447 (2)	0.77694 (12)	0.0245 (6)	
H38A	0.128124	0.397601	0.792713	0.029*	
H38B	0.168574	0.283730	0.795594	0.029*	
C39	0.1198 (3)	0.3308 (2)	0.72475 (12)	0.0234 (6)	
H39A	0.153892	0.385396	0.704552	0.028*	
H39B	0.011282	0.334617	0.724739	0.028*	
C40	0.1623 (3)	0.2341 (2)	0.70110 (12)	0.0224 (5)	
H40A	0.148970	0.179924	0.724353	0.027*	
H40B	0.096224	0.222071	0.672005	0.027*	
Cl1	0.9791 (2)	0.48793 (15)	0.42048 (6)	0.0278 (3)	0.8
O1	0.9439 (10)	0.4621 (8)	0.4702 (2)	0.0422 (15)	0.8
O2	1.1368 (6)	0.4904 (4)	0.4193 (2)	0.0509 (12)	0.8
O3	0.9212 (7)	0.5846 (4)	0.40984 (18)	0.0536 (13)	0.8
O4	0.9174 (5)	0.4175 (3)	0.38842 (15)	0.0544 (11)	0.8
Cl11	1.0220 (11)	0.4954 (7)	0.4308 (5)	0.060 (3)	0.2
O11	0.948 (3)	0.564 (3)	0.3972 (12)	0.122 (11)	0.2
O12	1.100 (3)	0.574 (2)	0.4601 (11)	0.124 (9)	0.2
O13	1.142 (3)	0.447 (2)	0.4147 (14)	0.097 (8)	0.2
O14	0.929 (4)	0.456 (3)	0.4636 (12)	0.051 (7)	0.2
Cl21	−0.06938 (19)	−0.00124 (11)	0.60601 (6)	0.0325 (3)	0.8
O21	−0.2031 (6)	−0.0381 (5)	0.58263 (18)	0.0585 (13)	0.8
O22	−0.0737 (6)	−0.0285 (4)	0.65718 (18)	0.0675 (14)	0.8
O23	−0.0640 (8)	0.1018 (3)	0.6043 (2)	0.0660 (15)	0.8
O24	0.0563 (6)	−0.0424 (4)	0.5862 (3)	0.0750 (17)	0.8
Cl31	−0.0636 (10)	−0.0010 (5)	0.5864 (3)	0.047 (2)	0.2
O31	−0.025 (3)	−0.0276 (12)	0.5420 (6)	0.059 (5)	0.2
O32	−0.067 (4)	−0.0860 (16)	0.6230 (8)	0.105 (10)	0.2
O33	0.036 (4)	0.062 (3)	0.6142 (10)	0.131 (14)	0.2
O34	−0.205 (3)	0.033 (3)	0.5921 (13)	0.135 (14)	0.2
O41	1.2346 (6)	0.5688 (3)	0.51620 (14)	0.0538 (11)	0.8
H41A	1.231686	0.522134	0.534874	0.081*	0.8
H41B	1.284936	0.549954	0.494184	0.081*	0.8
O42	−0.2372 (4)	0.0396 (3)	0.48350 (13)	0.0469 (10)	0.8
H42A	−0.211820	−0.017802	0.481961	0.070*	0.8
H42B	−0.260790	0.056688	0.510502	0.070*	0.8

### (µ-Tetrathioantimonato-κ2S:S')bis[(1,4,8,11-tetraazacyclotetradecane-κ4N)zinc(II)] perchlorate 0.8-hydrate Atomic displacement parameters (Å^2^)

**Table:**

	*U*^11^	*U*^22^	*U*^33^	*U*^12^	*U*^13^	*U*^23^
Sb1	0.01826 (7)	0.00984 (7)	0.01032 (7)	0.00212 (5)	0.00135 (6)	0.00076 (5)
S1	0.0178 (3)	0.0173 (3)	0.0196 (3)	0.0048 (2)	0.0041 (2)	0.0079 (2)
S2	0.0275 (3)	0.0110 (3)	0.0144 (3)	0.0004 (2)	0.0057 (2)	−0.0003 (2)
S3	0.0244 (3)	0.0135 (3)	0.0182 (3)	0.0028 (2)	−0.0022 (2)	0.0040 (2)
S4	0.0219 (3)	0.0176 (3)	0.0179 (3)	0.0010 (2)	−0.0022 (2)	−0.0038 (2)
Zn1	0.01847 (13)	0.01275 (13)	0.01295 (12)	0.00237 (10)	0.00118 (10)	−0.00002 (10)
N1	0.0250 (11)	0.0147 (10)	0.0158 (10)	0.0012 (8)	−0.0021 (8)	0.0003 (8)
C1	0.0326 (15)	0.0180 (13)	0.0147 (12)	−0.0001 (11)	−0.0042 (10)	−0.0004 (10)
C2	0.0352 (16)	0.0162 (12)	0.0154 (12)	0.0021 (11)	0.0028 (11)	−0.0032 (10)
N2	0.0244 (11)	0.0128 (9)	0.0161 (9)	0.0029 (8)	0.0025 (8)	0.0017 (8)
C3	0.0220 (13)	0.0212 (13)	0.0182 (12)	0.0051 (10)	0.0065 (10)	0.0041 (10)
C4	0.0201 (12)	0.0224 (13)	0.0226 (12)	0.0013 (10)	0.0031 (10)	0.0069 (11)
C5	0.0208 (12)	0.0193 (12)	0.0201 (12)	−0.0009 (10)	−0.0015 (10)	0.0014 (10)
N3	0.0212 (10)	0.0146 (10)	0.0152 (9)	0.0014 (8)	−0.0008 (8)	−0.0004 (8)
C6	0.0311 (14)	0.0159 (12)	0.0128 (11)	0.0004 (10)	0.0005 (10)	−0.0017 (9)
C7	0.0317 (15)	0.0151 (12)	0.0166 (12)	0.0054 (10)	0.0045 (10)	−0.0011 (10)
N4	0.0204 (10)	0.0141 (10)	0.0179 (10)	0.0050 (8)	0.0033 (8)	0.0019 (8)
C8	0.0203 (13)	0.0197 (13)	0.0256 (13)	0.0076 (10)	0.0065 (10)	0.0063 (11)
C9	0.0169 (12)	0.0224 (13)	0.0302 (14)	0.0028 (10)	0.0011 (10)	0.0081 (11)
C10	0.0205 (13)	0.0263 (14)	0.0262 (14)	0.0004 (11)	−0.0074 (11)	0.0037 (12)
Zn11	0.02547 (16)	0.01244 (12)	0.01717 (14)	−0.00326 (12)	0.00334 (12)	−0.00120 (12)
N11	0.0261 (12)	0.0296 (14)	0.0294 (13)	−0.0015 (10)	0.0052 (10)	−0.0042 (11)
C11	0.0244 (16)	0.058 (3)	0.0359 (18)	−0.0013 (16)	−0.0059 (13)	−0.0032 (18)
C12	0.031 (2)	0.065 (3)	0.052 (3)	−0.0160 (19)	−0.0003 (19)	−0.023 (2)
N12	0.0320 (14)	0.0356 (16)	0.0398 (16)	−0.0160 (12)	0.0103 (12)	−0.0172 (13)
C13	0.054 (3)	0.045 (3)	0.085 (4)	−0.034 (2)	0.037 (3)	−0.034 (3)
C14	0.074 (3)	0.0203 (16)	0.080 (3)	−0.0170 (18)	0.043 (3)	−0.019 (2)
C15	0.081 (3)	0.0159 (15)	0.073 (3)	−0.0015 (18)	0.045 (3)	0.0103 (18)
N13	0.0554 (19)	0.0177 (12)	0.0303 (14)	0.0067 (12)	0.0206 (13)	0.0048 (10)
C16	0.064 (3)	0.042 (2)	0.0206 (15)	0.0260 (19)	0.0149 (16)	0.0119 (15)
C17	0.044 (2)	0.049 (2)	0.0175 (14)	0.0166 (18)	0.0005 (13)	−0.0032 (15)
N14	0.0425 (15)	0.0224 (12)	0.0176 (11)	0.0062 (11)	0.0043 (10)	0.0004 (9)
C18	0.0399 (18)	0.0243 (15)	0.0263 (15)	0.0012 (13)	0.0026 (13)	−0.0120 (12)
C19	0.0458 (19)	0.0201 (14)	0.0300 (16)	0.0074 (13)	0.0093 (14)	−0.0047 (12)
C20	0.0348 (17)	0.0219 (14)	0.0352 (17)	0.0071 (12)	0.0104 (13)	0.0032 (13)
Sb11	0.02072 (8)	0.00981 (7)	0.01124 (7)	−0.00171 (5)	0.00407 (6)	0.00001 (5)
S11	0.0307 (3)	0.0108 (3)	0.0136 (3)	−0.0038 (2)	0.0085 (2)	−0.0006 (2)
S12	0.0189 (3)	0.0186 (3)	0.0294 (3)	−0.0020 (2)	0.0029 (2)	0.0104 (3)
S13	0.0217 (3)	0.0155 (3)	0.0148 (3)	−0.0025 (2)	0.0045 (2)	−0.0039 (2)
S14	0.0350 (4)	0.0149 (3)	0.0156 (3)	−0.0073 (3)	0.0039 (2)	−0.0043 (2)
Zn21	0.02385 (15)	0.01317 (14)	0.01295 (13)	0.00130 (12)	0.00185 (11)	−0.00026 (11)
N21	0.0318 (18)	0.0231 (16)	0.0174 (14)	0.0042 (13)	−0.0040 (13)	−0.0040 (13)
C21	0.057 (4)	0.034 (3)	0.015 (2)	0.013 (3)	−0.003 (2)	0.001 (2)
C22	0.065 (4)	0.026 (2)	0.0192 (19)	0.018 (2)	0.005 (2)	0.0080 (17)
N22	0.057 (3)	0.0171 (16)	0.0250 (18)	0.0042 (16)	0.0217 (17)	0.0026 (13)
C23	0.082 (6)	0.015 (2)	0.046 (5)	−0.003 (3)	0.037 (4)	0.001 (3)
C24	0.073 (4)	0.022 (2)	0.053 (4)	−0.019 (2)	0.037 (3)	−0.012 (2)
C25	0.039 (3)	0.043 (3)	0.048 (4)	−0.022 (3)	0.021 (3)	−0.026 (3)
N23	0.0263 (18)	0.039 (2)	0.0278 (18)	−0.0090 (16)	0.0112 (14)	−0.0160 (16)
C26	0.020 (2)	0.053 (3)	0.031 (3)	0.004 (2)	−0.0014 (18)	−0.019 (2)
C27	0.037 (5)	0.043 (4)	0.023 (3)	0.017 (3)	−0.003 (3)	0.000 (3)
N24	0.0255 (16)	0.0225 (17)	0.0187 (14)	0.0071 (13)	0.0052 (12)	0.0035 (12)
C28	0.039 (3)	0.0149 (19)	0.032 (2)	0.0069 (17)	0.011 (2)	0.0015 (17)
C29	0.033 (2)	0.0131 (17)	0.039 (2)	−0.0038 (15)	0.0124 (18)	−0.0070 (16)
C30	0.034 (3)	0.026 (2)	0.027 (2)	−0.001 (2)	0.0020 (19)	−0.0112 (18)
N21'	0.024 (4)	0.019 (4)	0.016 (4)	0.000 (3)	0.005 (3)	−0.001 (3)
C21'	0.020 (4)	0.026 (5)	0.026 (5)	−0.001 (4)	0.000 (4)	0.014 (4)
C22'	0.032 (6)	0.015 (6)	0.027 (7)	−0.002 (4)	0.015 (4)	0.010 (5)
N22'	0.024 (4)	0.019 (4)	0.023 (4)	0.000 (3)	0.013 (3)	0.000 (3)
C23'	0.040 (7)	0.022 (6)	0.027 (6)	−0.014 (5)	0.012 (4)	−0.006 (4)
C24'	0.024 (5)	0.049 (7)	0.031 (6)	0.000 (5)	−0.001 (4)	−0.017 (5)
C25'	0.009 (4)	0.057 (7)	0.025 (5)	−0.001 (5)	−0.007 (4)	−0.006 (5)
N23'	0.012 (3)	0.040 (5)	0.016 (4)	0.007 (3)	0.001 (3)	0.005 (3)
C26'	0.037 (12)	0.047 (8)	0.018 (6)	0.018 (6)	0.012 (6)	0.010 (5)
C27'	0.028 (5)	0.016 (5)	0.044 (7)	0.016 (4)	0.010 (5)	0.011 (5)
N24'	0.025 (4)	0.015 (3)	0.020 (3)	0.005 (3)	0.008 (3)	0.002 (3)
C28'	0.032 (6)	0.010 (4)	0.029 (5)	0.003 (4)	0.003 (4)	−0.010 (4)
C29'	0.028 (5)	0.029 (5)	0.019 (4)	−0.006 (4)	0.004 (4)	−0.003 (4)
C30'	0.031 (6)	0.026 (5)	0.017 (5)	−0.005 (4)	0.000 (4)	−0.003 (4)
Zn31	0.01959 (13)	0.01265 (13)	0.01706 (14)	−0.00204 (10)	0.00198 (10)	0.00103 (11)
N31	0.0248 (11)	0.0142 (10)	0.0187 (10)	−0.0026 (8)	0.0019 (9)	0.0020 (8)
C31	0.0336 (15)	0.0152 (12)	0.0185 (12)	−0.0028 (11)	−0.0007 (11)	0.0004 (10)
C32	0.0347 (16)	0.0168 (12)	0.0166 (12)	0.0021 (11)	0.0055 (11)	0.0008 (10)
N32	0.0245 (11)	0.0163 (10)	0.0168 (10)	0.0001 (8)	0.0040 (8)	0.0026 (8)
C33	0.0216 (13)	0.0235 (14)	0.0286 (14)	0.0031 (11)	0.0083 (11)	0.0059 (12)
C34	0.0218 (13)	0.0230 (14)	0.0290 (14)	0.0020 (10)	0.0030 (11)	0.0092 (12)
C35	0.0197 (13)	0.0240 (14)	0.0280 (14)	−0.0041 (10)	−0.0036 (11)	0.0069 (11)
N33	0.0224 (11)	0.0153 (10)	0.0215 (11)	−0.0034 (8)	−0.0004 (9)	0.0013 (9)
C36	0.0345 (16)	0.0180 (13)	0.0216 (13)	−0.0035 (11)	−0.0010 (12)	−0.0035 (11)
C37	0.0349 (16)	0.0170 (12)	0.0174 (13)	0.0003 (11)	0.0045 (11)	−0.0002 (10)
N34	0.0231 (11)	0.0139 (10)	0.0185 (10)	−0.0005 (8)	0.0044 (8)	0.0019 (8)
C38	0.0248 (14)	0.0222 (14)	0.0272 (14)	−0.0001 (11)	0.0085 (11)	0.0037 (11)
C39	0.0196 (12)	0.0214 (13)	0.0294 (14)	−0.0013 (10)	0.0020 (11)	0.0071 (12)
C40	0.0224 (13)	0.0182 (12)	0.0262 (13)	−0.0067 (10)	−0.0018 (10)	0.0053 (11)
Cl1	0.0324 (8)	0.0256 (6)	0.0256 (5)	0.0035 (5)	0.0025 (5)	−0.0043 (4)
O1	0.063 (4)	0.040 (3)	0.0241 (18)	0.005 (2)	0.0078 (18)	−0.0019 (17)
O2	0.036 (2)	0.068 (4)	0.049 (3)	0.008 (2)	0.0100 (18)	−0.009 (2)
O3	0.073 (4)	0.039 (2)	0.051 (2)	0.024 (2)	0.028 (2)	0.0175 (19)
O4	0.070 (3)	0.055 (3)	0.036 (2)	−0.009 (2)	−0.0166 (19)	−0.0176 (18)
Cl11	0.053 (5)	0.028 (3)	0.104 (9)	0.001 (3)	0.050 (5)	0.000 (4)
O11	0.075 (11)	0.14 (2)	0.157 (18)	0.044 (12)	0.082 (10)	0.080 (17)
O12	0.117 (14)	0.099 (13)	0.163 (17)	−0.068 (12)	0.084 (11)	−0.051 (12)
O13	0.069 (12)	0.081 (14)	0.15 (2)	0.024 (9)	0.067 (12)	−0.005 (11)
O14	0.034 (9)	0.038 (11)	0.083 (16)	−0.002 (7)	0.027 (9)	−0.001 (10)
Cl21	0.0414 (7)	0.0239 (5)	0.0325 (7)	−0.0003 (4)	0.0034 (6)	0.0029 (5)
O21	0.053 (3)	0.077 (4)	0.046 (2)	−0.021 (3)	0.007 (2)	0.001 (2)
O22	0.082 (4)	0.072 (3)	0.049 (3)	0.024 (3)	0.005 (2)	0.030 (2)
O23	0.109 (4)	0.0220 (18)	0.066 (3)	0.001 (2)	−0.003 (3)	0.0074 (19)
O24	0.065 (3)	0.053 (3)	0.111 (5)	−0.004 (2)	0.045 (3)	−0.014 (3)
Cl31	0.057 (4)	0.030 (3)	0.059 (5)	−0.014 (2)	0.038 (4)	−0.007 (3)
O31	0.107 (16)	0.028 (7)	0.046 (8)	−0.023 (8)	0.033 (9)	0.003 (6)
O32	0.21 (3)	0.061 (13)	0.049 (11)	−0.027 (14)	0.034 (16)	0.013 (9)
O33	0.17 (3)	0.14 (3)	0.078 (17)	−0.10 (3)	−0.025 (19)	0.012 (17)
O34	0.073 (13)	0.22 (4)	0.12 (3)	0.052 (19)	0.011 (15)	−0.03 (3)
O41	0.095 (3)	0.0326 (19)	0.0330 (18)	−0.015 (2)	−0.007 (2)	0.0099 (15)
O42	0.045 (2)	0.067 (3)	0.0295 (16)	−0.0248 (19)	0.0082 (15)	−0.0105 (17)

### (µ-Tetrathioantimonato-κ2S:S')bis[(1,4,8,11-tetraazacyclotetradecane-κ4N)zinc(II)] perchlorate 0.8-hydrate Geometric parameters (Å, º)

**Table:**

Sb1—S1	2.3409 (7)	C25—H25A	0.9900
Sb1—S2	2.3593 (7)	C25—H25B	0.9900
Sb1—S3	2.3090 (7)	C25—N23	1.485 (8)
Sb1—S4	2.3168 (7)	N23—H23	1.0000
S1—Zn1	2.4024 (7)	N23—C26	1.478 (8)
S2—Zn11	2.3694 (8)	C26—H26A	0.9900
Zn1—N1	2.137 (2)	C26—H26B	0.9900
Zn1—N2	2.118 (2)	C26—C27	1.530 (13)
Zn1—N3	2.144 (2)	C27—H27A	0.9900
Zn1—N4	2.116 (2)	C27—H27B	0.9900
N1—H1	1.0000	C27—N24	1.483 (10)
N1—C1	1.484 (4)	N24—H24	1.0000
N1—C10	1.485 (4)	N24—C28	1.484 (6)
C1—H1A	0.9900	C28—H28A	0.9900
C1—H1B	0.9900	C28—H28B	0.9900
C1—C2	1.527 (5)	C28—C29	1.514 (8)
C2—H2A	0.9900	C29—H29A	0.9900
C2—H2B	0.9900	C29—H29B	0.9900
C2—N2	1.471 (4)	C29—C30	1.521 (8)
N2—H2	1.0000	C30—H30A	0.9900
N2—C3	1.478 (4)	C30—H30B	0.9900
C3—H3A	0.9900	N21'—H21'	1.0000
C3—H3B	0.9900	N21'—C21'	1.475 (12)
C3—C4	1.525 (5)	N21'—C30'	1.463 (13)
C4—H4A	0.9900	C21'—H21C	0.9900
C4—H4B	0.9900	C21'—H21D	0.9900
C4—C5	1.529 (4)	C21'—C22'	1.498 (16)
C5—H5A	0.9900	C22'—H22C	0.9900
C5—H5B	0.9900	C22'—H22D	0.9900
C5—N3	1.479 (4)	C22'—N22'	1.438 (16)
N3—H3	1.0000	N22'—H22'	1.0000
N3—C6	1.471 (4)	N22'—C23'	1.494 (14)
C6—H6A	0.9900	C23'—H23C	0.9900
C6—H6B	0.9900	C23'—H23D	0.9900
C6—C7	1.524 (5)	C23'—C24'	1.522 (18)
C7—H7A	0.9900	C24'—H24C	0.9900
C7—H7B	0.9900	C24'—H24D	0.9900
C7—N4	1.483 (4)	C24'—C25'	1.523 (18)
N4—H4	1.0000	C25'—H25C	0.9900
N4—C8	1.487 (4)	C25'—H25D	0.9900
C8—H8A	0.9900	C25'—N23'	1.443 (15)
C8—H8B	0.9900	N23'—H23'	1.0000
C8—C9	1.525 (5)	N23'—C26'	1.52 (2)
C9—H9A	0.9900	C26'—H26C	0.9900
C9—H9B	0.9900	C26'—H26D	0.9900
C9—C10	1.525 (5)	C26'—C27'	1.51 (3)
C10—H10A	0.9900	C27'—H27C	0.9900
C10—H10B	0.9900	C27'—H27D	0.9900
Zn11—N11	2.107 (3)	C27'—N24'	1.467 (13)
Zn11—N12	2.124 (3)	N24'—H24'	1.0000
Zn11—N13	2.135 (3)	N24'—C28'	1.491 (13)
Zn11—N14	2.134 (3)	C28'—H28C	0.9900
N11—H11	1.0000	C28'—H28D	0.9900
N11—C11	1.484 (5)	C28'—C29'	1.535 (16)
N11—C20	1.473 (5)	C29'—H29C	0.9900
C11—H11A	0.9900	C29'—H29D	0.9900
C11—H11B	0.9900	C29'—C30'	1.516 (15)
C11—C12	1.522 (7)	C30'—H30C	0.9900
C12—H12A	0.9900	C30'—H30D	0.9900
C12—H12B	0.9900	Zn31—N31	2.114 (3)
C12—N12	1.472 (7)	Zn31—N32	2.141 (2)
N12—H12	1.0000	Zn31—N33	2.121 (3)
N12—C13	1.475 (6)	Zn31—N34	2.137 (2)
C13—H13A	0.9900	N31—H31	1.0000
C13—H13B	0.9900	N31—C31	1.479 (4)
C13—C14	1.511 (9)	N31—C40	1.488 (4)
C14—H14A	0.9900	C31—H31A	0.9900
C14—H14B	0.9900	C31—H31B	0.9900
C14—C15	1.521 (8)	C31—C32	1.533 (5)
C15—H15A	0.9900	C32—H32A	0.9900
C15—H15B	0.9900	C32—H32B	0.9900
C15—N13	1.475 (5)	C32—N32	1.476 (4)
N13—H13	1.0000	N32—H32	1.0000
N13—C16	1.509 (5)	N32—C33	1.480 (4)
C16—H16A	0.9900	C33—H33A	0.9900
C16—H16B	0.9900	C33—H33B	0.9900
C16—C17	1.503 (6)	C33—C34	1.522 (5)
C17—H17A	0.9900	C34—H34A	0.9900
C17—H17B	0.9900	C34—H34B	0.9900
C17—N14	1.469 (5)	C34—C35	1.525 (5)
N14—H14	1.0000	C35—H35A	0.9900
N14—C18	1.472 (4)	C35—H35B	0.9900
C18—H18A	0.9900	C35—N33	1.477 (4)
C18—H18B	0.9900	N33—H33	1.0000
C18—C19	1.513 (5)	N33—C36	1.480 (4)
C19—H19A	0.9900	C36—H36A	0.9900
C19—H19B	0.9900	C36—H36B	0.9900
C19—C20	1.529 (5)	C36—C37	1.522 (5)
C20—H20A	0.9900	C37—H37A	0.9900
C20—H20B	0.9900	C37—H37B	0.9900
Sb11—S11	2.3424 (7)	C37—N34	1.485 (4)
Sb11—S12	2.3572 (8)	N34—H34	1.0000
Sb11—S13	2.3030 (7)	N34—C38	1.486 (4)
Sb11—S14	2.3100 (7)	C38—H38A	0.9900
S11—Zn21	2.3806 (7)	C38—H38B	0.9900
S12—Zn31	2.3870 (8)	C38—C39	1.529 (5)
Zn21—N21	2.166 (4)	C39—H39A	0.9900
Zn21—N22	2.115 (4)	C39—H39B	0.9900
Zn21—N23	2.080 (4)	C39—C40	1.532 (5)
Zn21—N24	2.133 (3)	C40—H40A	0.9900
Zn21—N21'	2.143 (9)	C40—H40B	0.9900
Zn21—N22'	2.239 (9)	Cl1—O1	1.464 (6)
Zn21—N23'	2.144 (8)	Cl1—O2	1.439 (5)
Zn21—N24'	2.054 (8)	Cl1—O3	1.448 (5)
N21—H21	1.0000	Cl1—O4	1.403 (4)
N21—C21	1.472 (6)	Cl11—O11	1.46 (2)
N21—C30	1.475 (6)	Cl11—O12	1.50 (2)
C21—H21A	0.9900	Cl11—O13	1.375 (18)
C21—H21B	0.9900	Cl11—O14	1.38 (2)
C21—C22	1.510 (8)	Cl21—O21	1.440 (5)
C22—H22A	0.9900	Cl21—O22	1.458 (5)
C22—H22B	0.9900	Cl21—O23	1.411 (4)
C22—N22	1.470 (8)	Cl21—O24	1.410 (5)
N22—H22	1.0000	Cl31—O31	1.338 (15)
N22—C23	1.485 (8)	Cl31—O32	1.539 (18)
C23—H23A	0.9900	Cl31—O33	1.437 (19)
C23—H23B	0.9900	Cl31—O34	1.39 (2)
C23—C24	1.509 (12)	O41—H41A	0.8206
C24—H24A	0.9900	O41—H41B	0.8199
C24—H24B	0.9900	O42—H42A	0.8197
C24—C25	1.513 (11)	O42—H42B	0.8174
			
S1—Sb1—S2	104.87 (2)	C23—C24—C25	116.7 (6)
S3—Sb1—S1	113.73 (3)	H24A—C24—H24B	107.3
S3—Sb1—S2	106.93 (3)	C25—C24—H24A	108.1
S3—Sb1—S4	109.83 (3)	C25—C24—H24B	108.1
S4—Sb1—S1	107.25 (3)	C24—C25—H25A	109.2
S4—Sb1—S2	114.30 (3)	C24—C25—H25B	109.2
Sb1—S1—Zn1	105.77 (3)	H25A—C25—H25B	107.9
Sb1—S2—Zn11	105.00 (3)	N23—C25—C24	111.9 (5)
N1—Zn1—S1	105.40 (7)	N23—C25—H25A	109.2
N1—Zn1—N3	152.59 (9)	N23—C25—H25B	109.2
N2—Zn1—S1	97.91 (7)	Zn21—N23—H23	108.3
N2—Zn1—N1	82.77 (10)	C25—N23—Zn21	113.7 (4)
N2—Zn1—N3	87.19 (9)	C25—N23—H23	108.3
N3—Zn1—S1	101.20 (7)	C26—N23—Zn21	103.0 (3)
N4—Zn1—S1	109.81 (7)	C26—N23—C25	114.9 (5)
N4—Zn1—N1	94.64 (10)	C26—N23—H23	108.3
N4—Zn1—N2	151.76 (9)	N23—C26—H26A	110.2
N4—Zn1—N3	82.36 (9)	N23—C26—H26B	110.2
Zn1—N1—H1	105.6	N23—C26—C27	107.6 (6)
C1—N1—Zn1	107.45 (19)	H26A—C26—H26B	108.5
C1—N1—H1	105.6	C27—C26—H26A	110.2
C1—N1—C10	112.3 (2)	C27—C26—H26B	110.2
C10—N1—Zn1	119.28 (19)	C26—C27—H27A	109.4
C10—N1—H1	105.6	C26—C27—H27B	109.4
N1—C1—H1A	109.6	H27A—C27—H27B	108.0
N1—C1—H1B	109.6	N24—C27—C26	111.3 (8)
N1—C1—C2	110.4 (2)	N24—C27—H27A	109.4
H1A—C1—H1B	108.1	N24—C27—H27B	109.4
C2—C1—H1A	109.6	Zn21—N24—H24	106.8
C2—C1—H1B	109.6	C27—N24—Zn21	106.3 (5)
C1—C2—H2A	110.2	C27—N24—H24	106.8
C1—C2—H2B	110.2	C27—N24—C28	111.8 (6)
H2A—C2—H2B	108.5	C28—N24—Zn21	117.7 (3)
N2—C2—C1	107.4 (2)	C28—N24—H24	106.8
N2—C2—H2A	110.2	N24—C28—H28A	109.0
N2—C2—H2B	110.2	N24—C28—H28B	109.0
Zn1—N2—H2	108.1	N24—C28—C29	113.1 (4)
C2—N2—Zn1	104.70 (18)	H28A—C28—H28B	107.8
C2—N2—H2	108.1	C29—C28—H28A	109.0
C2—N2—C3	113.8 (2)	C29—C28—H28B	109.0
C3—N2—Zn1	113.79 (17)	C28—C29—H29A	108.6
C3—N2—H2	108.1	C28—C29—H29B	108.6
N2—C3—H3A	109.3	C28—C29—C30	114.6 (4)
N2—C3—H3B	109.3	H29A—C29—H29B	107.6
N2—C3—C4	111.5 (2)	C30—C29—H29A	108.6
H3A—C3—H3B	108.0	C30—C29—H29B	108.6
C4—C3—H3A	109.3	N21—C30—C29	112.7 (4)
C4—C3—H3B	109.3	N21—C30—H30A	109.0
C3—C4—H4A	108.2	N21—C30—H30B	109.0
C3—C4—H4B	108.2	C29—C30—H30A	109.0
C3—C4—C5	116.2 (2)	C29—C30—H30B	109.0
H4A—C4—H4B	107.4	H30A—C30—H30B	107.8
C5—C4—H4A	108.2	Zn21—N21'—H21'	104.8
C5—C4—H4B	108.2	C21'—N21'—Zn21	109.4 (6)
C4—C5—H5A	109.3	C21'—N21'—H21'	104.8
C4—C5—H5B	109.3	C30'—N21'—Zn21	118.7 (7)
H5A—C5—H5B	108.0	C30'—N21'—H21'	104.8
N3—C5—C4	111.5 (2)	C30'—N21'—C21'	112.9 (8)
N3—C5—H5A	109.3	N21'—C21'—H21C	109.3
N3—C5—H5B	109.3	N21'—C21'—H21D	109.3
Zn1—N3—H3	108.0	N21'—C21'—C22'	111.7 (10)
C5—N3—Zn1	114.74 (18)	H21C—C21'—H21D	107.9
C5—N3—H3	108.0	C22'—C21'—H21C	109.3
C6—N3—Zn1	103.49 (18)	C22'—C21'—H21D	109.3
C6—N3—C5	114.3 (2)	C21'—C22'—H22C	109.9
C6—N3—H3	108.0	C21'—C22'—H22D	109.9
N3—C6—H6A	110.2	H22C—C22'—H22D	108.3
N3—C6—H6B	110.2	N22'—C22'—C21'	109.1 (11)
N3—C6—C7	107.6 (2)	N22'—C22'—H22C	109.9
H6A—C6—H6B	108.5	N22'—C22'—H22D	109.9
C7—C6—H6A	110.2	Zn21—N22'—H22'	108.1
C7—C6—H6B	110.2	C22'—N22'—Zn21	102.9 (8)
C6—C7—H7A	109.7	C22'—N22'—H22'	108.1
C6—C7—H7B	109.7	C22'—N22'—C23'	113.7 (10)
H7A—C7—H7B	108.2	C23'—N22'—Zn21	115.6 (7)
N4—C7—C6	109.9 (2)	C23'—N22'—H22'	108.1
N4—C7—H7A	109.7	N22'—C23'—H23C	109.4
N4—C7—H7B	109.7	N22'—C23'—H23D	109.4
Zn1—N4—H4	104.8	N22'—C23'—C24'	111.0 (10)
C7—N4—Zn1	108.74 (18)	H23C—C23'—H23D	108.0
C7—N4—H4	104.8	C24'—C23'—H23C	109.4
C7—N4—C8	112.4 (2)	C24'—C23'—H23D	109.4
C8—N4—Zn1	119.90 (18)	C23'—C24'—H24C	107.8
C8—N4—H4	104.8	C23'—C24'—H24D	107.8
N4—C8—H8A	109.4	C23'—C24'—C25'	118.0 (11)
N4—C8—H8B	109.4	H24C—C24'—H24D	107.2
N4—C8—C9	111.4 (2)	C25'—C24'—H24C	107.8
H8A—C8—H8B	108.0	C25'—C24'—H24D	107.8
C9—C8—H8A	109.4	C24'—C25'—H25C	109.5
C9—C8—H8B	109.4	C24'—C25'—H25D	109.5
C8—C9—H9A	108.6	H25C—C25'—H25D	108.1
C8—C9—H9B	108.6	N23'—C25'—C24'	110.8 (10)
C8—C9—C10	114.8 (3)	N23'—C25'—H25C	109.5
H9A—C9—H9B	107.5	N23'—C25'—H25D	109.5
C10—C9—H9A	108.6	Zn21—N23'—H23'	109.1
C10—C9—H9B	108.6	C25'—N23'—Zn21	114.0 (7)
N1—C10—C9	111.9 (2)	C25'—N23'—H23'	109.1
N1—C10—H10A	109.2	C25'—N23'—C26'	114.0 (13)
N1—C10—H10B	109.2	C26'—N23'—Zn21	101.3 (11)
C9—C10—H10A	109.2	C26'—N23'—H23'	109.1
C9—C10—H10B	109.2	N23'—C26'—H26C	109.8
H10A—C10—H10B	107.9	N23'—C26'—H26D	109.8
N11—Zn11—S2	100.54 (8)	H26C—C26'—H26D	108.2
N11—Zn11—N12	83.40 (13)	C27'—C26'—N23'	109.4 (17)
N11—Zn11—N13	152.68 (12)	C27'—C26'—H26C	109.8
N11—Zn11—N14	89.63 (11)	C27'—C26'—H26D	109.8
N12—Zn11—S2	105.03 (9)	C26'—C27'—H27C	109.1
N12—Zn11—N13	93.99 (14)	C26'—C27'—H27D	109.1
N12—Zn11—N14	155.29 (12)	H27C—C27'—H27D	107.8
N13—Zn11—S2	106.37 (9)	N24'—C27'—C26'	112.5 (15)
N14—Zn11—S2	99.53 (9)	N24'—C27'—H27C	109.1
N14—Zn11—N13	81.45 (12)	N24'—C27'—H27D	109.1
Zn11—N11—H11	109.0	Zn21—N24'—H24'	105.9
C11—N11—Zn11	103.1 (2)	C27'—N24'—Zn21	109.0 (7)
C11—N11—H11	109.0	C27'—N24'—H24'	105.9
C20—N11—Zn11	114.4 (2)	C27'—N24'—C28'	111.6 (9)
C20—N11—H11	109.0	C28'—N24'—Zn21	117.8 (6)
C20—N11—C11	112.2 (3)	C28'—N24'—H24'	105.9
N11—C11—H11A	109.8	N24'—C28'—H28C	109.0
N11—C11—H11B	109.8	N24'—C28'—H28D	109.0
N11—C11—C12	109.3 (4)	N24'—C28'—C29'	112.8 (8)
H11A—C11—H11B	108.3	H28C—C28'—H28D	107.8
C12—C11—H11A	109.8	C29'—C28'—H28C	109.0
C12—C11—H11B	109.8	C29'—C28'—H28D	109.0
C11—C12—H12A	109.2	C28'—C29'—H29C	108.8
C11—C12—H12B	109.2	C28'—C29'—H29D	108.8
H12A—C12—H12B	107.9	H29C—C29'—H29D	107.7
N12—C12—C11	112.1 (3)	C30'—C29'—C28'	113.6 (10)
N12—C12—H12A	109.2	C30'—C29'—H29C	108.8
N12—C12—H12B	109.2	C30'—C29'—H29D	108.8
Zn11—N12—H12	105.5	N21'—C30'—C29'	113.5 (9)
C12—N12—Zn11	108.3 (3)	N21'—C30'—H30C	108.9
C12—N12—H12	105.5	N21'—C30'—H30D	108.9
C12—N12—C13	113.2 (4)	C29'—C30'—H30C	108.9
C13—N12—Zn11	117.8 (3)	C29'—C30'—H30D	108.9
C13—N12—H12	105.5	H30C—C30'—H30D	107.7
N12—C13—H13A	108.9	N31—Zn31—S12	108.13 (7)
N12—C13—H13B	108.9	N31—Zn31—N32	83.03 (10)
N12—C13—C14	113.5 (4)	N31—Zn31—N33	153.04 (9)
H13A—C13—H13B	107.7	N31—Zn31—N34	94.57 (10)
C14—C13—H13A	108.9	N32—Zn31—S12	100.50 (7)
C14—C13—H13B	108.9	N33—Zn31—S12	98.35 (7)
C13—C14—H14A	108.8	N33—Zn31—N32	87.34 (10)
C13—C14—H14B	108.8	N33—Zn31—N34	82.96 (10)
C13—C14—C15	114.0 (4)	N34—Zn31—S12	105.38 (7)
H14A—C14—H14B	107.7	N34—Zn31—N32	153.40 (9)
C15—C14—H14A	108.8	Zn31—N31—H31	105.2
C15—C14—H14B	108.8	C31—N31—Zn31	108.09 (19)
C14—C15—H15A	109.2	C31—N31—H31	105.2
C14—C15—H15B	109.2	C31—N31—C40	111.9 (2)
H15A—C15—H15B	107.9	C40—N31—Zn31	119.95 (19)
N13—C15—C14	111.9 (3)	C40—N31—H31	105.2
N13—C15—H15A	109.2	N31—C31—H31A	109.8
N13—C15—H15B	109.2	N31—C31—H31B	109.8
Zn11—N13—H13	106.7	N31—C31—C32	109.5 (3)
C15—N13—Zn11	115.2 (3)	H31A—C31—H31B	108.2
C15—N13—H13	106.7	C32—C31—H31A	109.8
C15—N13—C16	111.9 (3)	C32—C31—H31B	109.8
C16—N13—Zn11	109.0 (2)	C31—C32—H32A	110.3
C16—N13—H13	106.7	C31—C32—H32B	110.3
N13—C16—H16A	109.8	H32A—C32—H32B	108.5
N13—C16—H16B	109.8	N32—C32—C31	107.2 (2)
H16A—C16—H16B	108.2	N32—C32—H32A	110.3
C17—C16—N13	109.4 (3)	N32—C32—H32B	110.3
C17—C16—H16A	109.8	Zn31—N32—H32	108.5
C17—C16—H16B	109.8	C32—N32—Zn31	102.91 (18)
C16—C17—H17A	110.0	C32—N32—H32	108.5
C16—C17—H17B	110.0	C32—N32—C33	114.1 (2)
H17A—C17—H17B	108.4	C33—N32—Zn31	113.92 (19)
N14—C17—C16	108.5 (4)	C33—N32—H32	108.5
N14—C17—H17A	110.0	N32—C33—H33A	109.4
N14—C17—H17B	110.0	N32—C33—H33B	109.4
Zn11—N14—H14	109.3	N32—C33—C34	111.4 (2)
C17—N14—Zn11	103.6 (2)	H33A—C33—H33B	108.0
C17—N14—H14	109.3	C34—C33—H33A	109.4
C17—N14—C18	113.9 (3)	C34—C33—H33B	109.4
C18—N14—Zn11	111.4 (2)	C33—C34—H34A	108.1
C18—N14—H14	109.3	C33—C34—H34B	108.1
N14—C18—H18A	109.1	C33—C34—C35	116.7 (3)
N14—C18—H18B	109.1	H34A—C34—H34B	107.3
N14—C18—C19	112.3 (3)	C35—C34—H34A	108.1
H18A—C18—H18B	107.9	C35—C34—H34B	108.1
C19—C18—H18A	109.1	C34—C35—H35A	109.3
C19—C18—H18B	109.1	C34—C35—H35B	109.3
C18—C19—H19A	108.3	H35A—C35—H35B	108.0
C18—C19—H19B	108.3	N33—C35—C34	111.5 (3)
C18—C19—C20	116.0 (3)	N33—C35—H35A	109.3
H19A—C19—H19B	107.4	N33—C35—H35B	109.3
C20—C19—H19A	108.3	Zn31—N33—H33	108.5
C20—C19—H19B	108.3	C35—N33—Zn31	113.57 (19)
N11—C20—C19	112.0 (3)	C35—N33—H33	108.5
N11—C20—H20A	109.2	C35—N33—C36	113.7 (2)
N11—C20—H20B	109.2	C36—N33—Zn31	103.70 (19)
C19—C20—H20A	109.2	C36—N33—H33	108.5
C19—C20—H20B	109.2	N33—C36—H36A	110.3
H20A—C20—H20B	107.9	N33—C36—H36B	110.3
S11—Sb11—S12	107.21 (3)	N33—C36—C37	107.3 (3)
S13—Sb11—S11	107.98 (3)	H36A—C36—H36B	108.5
S13—Sb11—S12	107.65 (3)	C37—C36—H36A	110.3
S13—Sb11—S14	116.20 (3)	C37—C36—H36B	110.3
S14—Sb11—S11	109.08 (3)	C36—C37—H37A	109.8
S14—Sb11—S12	108.37 (3)	C36—C37—H37B	109.8
Sb11—S11—Zn21	107.43 (3)	H37A—C37—H37B	108.2
Sb11—S12—Zn31	104.62 (3)	N34—C37—C36	109.6 (3)
N21—Zn21—S11	105.56 (10)	N34—C37—H37A	109.8
N22—Zn21—S11	97.01 (11)	N34—C37—H37B	109.8
N22—Zn21—N21	81.30 (17)	Zn31—N34—H34	105.8
N22—Zn21—N24	154.77 (14)	C37—N34—Zn31	107.20 (19)
N23—Zn21—S11	99.93 (10)	C37—N34—H34	105.8
N23—Zn21—N21	154.15 (14)	C37—N34—C38	112.0 (2)
N23—Zn21—N22	91.52 (19)	C38—N34—Zn31	119.29 (19)
N23—Zn21—N24	84.13 (17)	C38—N34—H34	105.8
N24—Zn21—S11	108.22 (9)	N34—C38—H38A	109.3
N24—Zn21—N21	91.85 (14)	N34—C38—H38B	109.3
N21'—Zn21—S11	97.5 (2)	N34—C38—C39	111.8 (2)
N21'—Zn21—N22'	80.1 (3)	H38A—C38—H38B	107.9
N21'—Zn21—N23'	149.3 (3)	C39—C38—H38A	109.3
N22'—Zn21—S11	94.2 (2)	C39—C38—H38B	109.3
N23'—Zn21—S11	109.6 (2)	C38—C39—H39A	108.6
N23'—Zn21—N22'	83.6 (3)	C38—C39—H39B	108.6
N24'—Zn21—S11	115.3 (2)	C38—C39—C40	114.5 (3)
N24'—Zn21—N21'	96.3 (3)	H39A—C39—H39B	107.6
N24'—Zn21—N22'	150.4 (3)	C40—C39—H39A	108.6
N24'—Zn21—N23'	85.3 (4)	C40—C39—H39B	108.6
Zn21—N21—H21	105.9	N31—C40—C39	111.8 (2)
C21—N21—Zn21	107.9 (3)	N31—C40—H40A	109.3
C21—N21—H21	105.9	N31—C40—H40B	109.3
C21—N21—C30	111.2 (4)	C39—C40—H40A	109.3
C30—N21—Zn21	119.2 (3)	C39—C40—H40B	109.3
C30—N21—H21	105.9	H40A—C40—H40B	107.9
N21—C21—H21A	109.4	O2—Cl1—O1	107.0 (5)
N21—C21—H21B	109.4	O2—Cl1—O3	109.2 (4)
N21—C21—C22	111.1 (4)	O3—Cl1—O1	108.3 (5)
H21A—C21—H21B	108.0	O4—Cl1—O1	108.7 (5)
C22—C21—H21A	109.4	O4—Cl1—O2	111.8 (3)
C22—C21—H21B	109.4	O4—Cl1—O3	111.6 (3)
C21—C22—H22A	110.2	O11—Cl11—O12	94 (2)
C21—C22—H22B	110.2	O13—Cl11—O11	117 (2)
H22A—C22—H22B	108.5	O13—Cl11—O12	98.8 (19)
N22—C22—C21	107.3 (4)	O13—Cl11—O14	123 (2)
N22—C22—H22A	110.2	O14—Cl11—O11	113 (2)
N22—C22—H22B	110.2	O14—Cl11—O12	103 (2)
Zn21—N22—H22	108.6	O21—Cl21—O22	106.1 (3)
C22—N22—Zn21	104.5 (3)	O23—Cl21—O21	111.4 (4)
C22—N22—H22	108.6	O23—Cl21—O22	106.8 (4)
C22—N22—C23	114.6 (5)	O24—Cl21—O21	111.9 (4)
C23—N22—Zn21	111.8 (4)	O24—Cl21—O22	109.4 (4)
C23—N22—H22	108.6	O24—Cl21—O23	110.9 (4)
N22—C23—H23A	109.3	O31—Cl31—O32	114.0 (13)
N22—C23—H23B	109.3	O31—Cl31—O33	117.4 (17)
N22—C23—C24	111.4 (6)	O31—Cl31—O34	119 (2)
H23A—C23—H23B	108.0	O33—Cl31—O32	98.0 (18)
C24—C23—H23A	109.3	O34—Cl31—O32	97 (2)
C24—C23—H23B	109.3	O34—Cl31—O33	108 (2)
C23—C24—H24A	108.1	H41A—O41—H41B	104.7
C23—C24—H24B	108.1	H42A—O42—H42B	114.1
			
Zn1—N1—C1—C2	30.6 (3)	C21—N21—C30—C29	177.4 (5)
Zn1—N1—C10—C9	−45.9 (3)	C21—C22—N22—Zn21	−54.1 (4)
Zn1—N2—C3—C4	−66.3 (3)	C21—C22—N22—C23	−176.8 (5)
Zn1—N3—C6—C7	−52.6 (2)	C22—N22—C23—C24	−178.1 (5)
Zn1—N4—C8—C9	47.0 (3)	N22—C23—C24—C25	−70.8 (8)
N1—C1—C2—N2	−56.6 (3)	C23—C24—C25—N23	68.6 (7)
C1—N1—C10—C9	−172.9 (3)	C24—C25—N23—Zn21	−60.6 (5)
C1—C2—N2—Zn1	51.4 (3)	C24—C25—N23—C26	−179.0 (5)
C1—C2—N2—C3	176.3 (2)	C25—N23—C26—C27	177.8 (6)
C2—N2—C3—C4	173.8 (2)	N23—C26—C27—N24	−54.8 (9)
N2—C3—C4—C5	67.7 (3)	C26—C27—N24—Zn21	25.2 (8)
C3—C4—C5—N3	−65.8 (3)	C26—C27—N24—C28	154.9 (6)
C4—C5—N3—Zn1	63.0 (3)	C27—N24—C28—C29	−178.8 (5)
C4—C5—N3—C6	−177.6 (2)	N24—C28—C29—C30	74.3 (5)
C5—N3—C6—C7	−178.1 (2)	C28—C29—C30—N21	−71.3 (5)
N3—C6—C7—N4	56.5 (3)	C30—N21—C21—C22	−158.6 (5)
C6—C7—N4—Zn1	−28.9 (3)	N21'—C21'—C22'—N22'	55.8 (15)
C6—C7—N4—C8	−164.1 (2)	C21'—N21'—C30'—C29'	173.7 (10)
C7—N4—C8—C9	176.5 (2)	C21'—C22'—N22'—Zn21	−52.1 (12)
N4—C8—C9—C10	−75.8 (3)	C21'—C22'—N22'—C23'	−177.9 (11)
C8—C9—C10—N1	75.5 (3)	C22'—N22'—C23'—C24'	178.8 (11)
C10—N1—C1—C2	163.6 (3)	N22'—C23'—C24'—C25'	−62.2 (14)
Zn11—N11—C11—C12	51.1 (3)	C23'—C24'—C25'—N23'	68.6 (14)
Zn11—N11—C20—C19	−61.3 (3)	C24'—C25'—N23'—Zn21	−71.9 (10)
Zn11—N12—C13—C14	−49.6 (5)	C24'—C25'—N23'—C26'	172.5 (16)
Zn11—N13—C16—C17	−24.3 (4)	C25'—N23'—C26'—C27'	170.2 (15)
Zn11—N14—C18—C19	65.6 (3)	N23'—C26'—C27'—N24'	−51 (2)
N11—C11—C12—N12	−49.4 (5)	C26'—C27'—N24'—Zn21	24.7 (15)
C11—N11—C20—C19	−178.3 (3)	C26'—C27'—N24'—C28'	156.4 (14)
C11—C12—N12—Zn11	20.0 (5)	C27'—N24'—C28'—C29'	−177.4 (9)
C11—C12—N12—C13	152.6 (4)	N24'—C28'—C29'—C30'	76.3 (12)
C12—N12—C13—C14	−177.3 (4)	C28'—C29'—C30'—N21'	−72.3 (13)
N12—C13—C14—C15	72.4 (5)	C30'—N21'—C21'—C22'	−161.3 (11)
C13—C14—C15—N13	−77.5 (5)	Zn31—N31—C31—C32	−31.4 (3)
C14—C15—N13—Zn11	57.5 (4)	Zn31—N31—C40—C39	46.8 (3)
C14—C15—N13—C16	−177.3 (4)	Zn31—N32—C33—C34	64.0 (3)
C15—N13—C16—C17	−152.9 (4)	Zn31—N33—C36—C37	52.6 (3)
N13—C16—C17—N14	54.2 (4)	Zn31—N34—C38—C39	−47.1 (3)
C16—C17—N14—Zn11	−55.1 (3)	N31—C31—C32—N32	58.6 (3)
C16—C17—N14—C18	−176.3 (3)	C31—N31—C40—C39	174.9 (2)
C17—N14—C18—C19	−177.6 (3)	C31—C32—N32—Zn31	−52.7 (2)
N14—C18—C19—C20	−70.1 (4)	C31—C32—N32—C33	−176.6 (2)
C18—C19—C20—N11	66.4 (4)	C32—N32—C33—C34	−178.2 (3)
C20—N11—C11—C12	174.7 (3)	N32—C33—C34—C35	−66.2 (4)
Zn21—N21—C21—C22	−26.1 (6)	C33—C34—C35—N33	67.1 (4)
Zn21—N21—C30—C29	51.0 (5)	C34—C35—N33—Zn31	−65.5 (3)
Zn21—N22—C23—C24	63.2 (7)	C34—C35—N33—C36	176.2 (3)
Zn21—N23—C26—C27	53.6 (6)	C35—N33—C36—C37	176.5 (3)
Zn21—N24—C28—C29	−55.3 (5)	N33—C36—C37—N34	−58.9 (3)
Zn21—N21'—C21'—C22'	−26.7 (12)	C36—C37—N34—Zn31	32.4 (3)
Zn21—N21'—C30'—C29'	43.7 (13)	C36—C37—N34—C38	165.0 (3)
Zn21—N22'—C23'—C24'	60.1 (12)	C37—N34—C38—C39	−173.5 (3)
Zn21—N23'—C26'—C27'	47 (2)	N34—C38—C39—C40	75.5 (3)
Zn21—N24'—C28'—C29'	−50.3 (11)	C38—C39—C40—N31	−75.1 (3)
N21—C21—C22—N22	55.0 (6)	C40—N31—C31—C32	−165.6 (2)

### (µ-Tetrathioantimonato-κ2S:S')bis[(1,4,8,11-tetraazacyclotetradecane-κ4N)zinc(II)] perchlorate 0.8-hydrate Hydrogen-bond geometry (Å, º)

**Table:**

*D*—H···*A*	*D*—H	H···*A*	*D*···*A*	*D*—H···*A*
N4—H4···S2	1.00	2.30	3.289 (2)	172
N11—H11···O42	1.00	2.00	2.948 (5)	157
N12—H12···S4	1.00	2.40	3.404 (3)	178
N13—H13···S3	1.00	2.46	3.444 (3)	169
N14—H14···O24	1.00	2.17	3.135 (6)	163
N14—H14···O31	1.00	2.28	3.115 (19)	140
N14—H14···O33	1.00	2.47	3.41 (4)	156
N22—H22···O3	1.00	2.16	3.158 (6)	175
N23—H23···O41	1.00	2.35	3.235 (6)	147
N24—H24···S12	1.00	2.56	3.483 (3)	154
N22′—H22′···O12	1.00	2.29	3.27 (3)	166
N31—H31···S14	1.00	2.36	3.355 (3)	174
N33—H33···S3^i^	1.00	2.53	3.419 (3)	148
N34—H34···S13	1.00	2.58	3.532 (2)	159
O41—H41*A*···S14^ii^	0.82	2.48	3.256 (4)	158

Symmetry codes: (i) *x*, −*y*, *z*+1/2; (ii) *x*+1, *y*, *z*.
